# Nano-γ-Al_2_O_3_/SbCl_5_: an efficient catalyst for the synthesis of 2,3-dihydroperimidines†

**DOI:** 10.1039/c7ra13593a

**Published:** 2018-02-07

**Authors:** Abdolhamid Bamoniri, Bi Bi Fatemeh Mirjalili, Sepideh Saleh

**Affiliations:** Departement of Org. Chem., Faculty of Chem., University of Kashan Kashan I. R. Iran bamoniri@kashanu.ac.ir; Department of Chem., College of Sci., Yazd University Yazd I. R. Iran

## Abstract

Nano-γ-Al_2_O_3_/SbCl_5_ as a new Lewis acid nano catalyst was synthesized and characterized by FTIR, XRD, FESEM, TEM, EDS, BET and TGA techniques. Nano-γ-Al_2_O_3_/SbCl_5_ has been employed for synthesis of 2-substituted perimidines *via* reaction of naphthalene-1,8-diamine with various aldehydes at room temperature under solvent-free conditions. This protocol proffers several benefits including high yields, easy workup, short reaction times and simple reaction conditions.

## Abstract 2

Nano-γ-Al_2_O_3_/SbCl_5_ as a new Lewis acid nano catalyst was synthesized and characterized by FTIR, XRD, FESEM, TEM, EDS, BET and TGA techniques.

## Graphical abstract

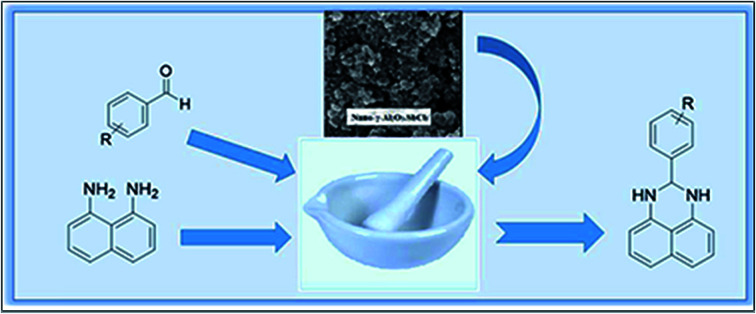

## 1. Introduction

Perimidines exhibit a diverse range of biological activities; such as antibacterial, antifungal, anti-inflammatory and antitumor.^1–6^ It was of interest to explore the suitability of some perimidines derivatives as potential DNA-intercalating ligands.^7^ A synthetic method for the preparation of perimidines is the condensation reaction of 1,8-diaminonaphthalene with various carbonyl groups.^8–10^ However, most of these methods suffers of significant side reaction, low yield and have cumbersome work-up procedures.

Some catalysts are reported for perimidine synthesis such as zeolite,^11^ CMK-5-SO_3_H,^12^ BiCl_3_,^13^ BF_3_·H_2_O,^14^ Yb(OTf)_3_,^15^ Cu(NO_3_)_2_·6H_2_O,^16^ FePO_4_,^17^ Fe_3_O_4_/SiO_2_/(CH_2_)_3_N^+^Me_3_Br_3_^−^,^18^ [BTBA]Cl–FeCl_3_,^19^ nano-silica sulfuric acid,^20^ amberlyst 15 (ref. 21) and molecular iodine.^22^

Antimony pentachloride (SbCl_5_), a thin and fuming liquid, is applied in industry and organic synthesis. Where of, antimony pentachloride is a liquid with a great specific gravity that fumigates in air and reacts with the humidity to form HCl, the tactility and the usability of SbCl_5_ as a liquid form is arduous and the supported form is really preferable. It has been acclaimed that the supported SbCl_5_ is a solid superacid. SbCl_5_ is used immensely in organic synthesis as a Lewis acid for elevating a variety of organic reactions.^23^ Solid-acid catalysts are commonly classified by their Brønsted and/or Lewis acidity, the intensity and number of these positions, and the morphology of the support. The synthesis of net Brønsted and net Lewis acid catalysts attracts a major degree of academic concern.^24^ Alumina (Al_2_O_3_) is applied both as a catalyst for divers types of reaction and as a support for metals. Al_2_O_3_ is very repeatedly applied as a support of industrial divers' types of reaction and as a support for metals. Al_2_O_3_ is very repeatedly applied as a support of catalysts for its mechanical intensity also its potent interaction with metals and metal oxides that provides high propagation of the supported compounds. As for the surface properties,  alumina is commonly considered as acidic rather than basic,  but basic positions coexist.^25^ Alumina is a main material for usages in ultrafiltration of salts, as an automobile exhaust catalyst, and in petroleum purification. Porous γ-alumina with equal channels, high surface area, and slender pore-size repartition possesses conjunction better physicochemical properties. However, the manufacturing of ordered and thermally constant porous alumina is demonstrated due to its susceptibility for hydrolysis and phase transition-induced demolition of ordered pore structure.^26^

Here in, we wish to report a simple method for the synthesis of nano-γ-Al_2_O_3_/SbCl_5_ and its usage in the synthesis of 2,3-dihydroperimidines under solvent-free grinding condition at room temperature.

## 2. Experimental

### 2.1 Material and methods

All compounds were purchased from Fluka and Merck chemical company and used without any additional purification. Fourier transform infrared (FT-IR) spectra were run on a Nicolet Magna 550 spectrometer. A Bruker (DRX-400 Avance) NMR was used to record the ^1^H-NMR and ^13^C-NMR spectra. XRD pattern using Philips Xpert MP diffractometer (Cu Kα, radiation, *k* = 0.154056 nm) was achieved. FE-SEM was obtained on a Mira Tescan. Transmission electron microscope (TEM) was recorded on a Philips-CM 120-with LaB_6_ cathode instrument on an accelerating voltage of 120 kV. The thermal gravimetric analysis (TGA) was done with “STA 504” instrument. Energy-dispersive X-ray spectroscopy (EDS) of SbCl_5_/nano-γ-Al_2_O_3_ was measured by EDS instrument, Phenom pro X. BET surface area analysis of catalyst was done with Micrometrics, Tristar II 3020 analyser.

### 2.2 Preparation of nano-γ-Al_2_O_3_

NaOH (600 ml, 1 M), was added drop-wise to a slurry containing Al_2_(SO_4_)_3_·18H_2_O (66 g). The mixture was stirred at room temperature. The resulted suspension was filtered to obtain the white solid Al(OH)_3_. Then solid were washed with distilled water until no more sulfate ions were detected in the washings. Following the aging step, NaOH (100 ml, 1 M) was added to a beaker containing Al(OH)_3_ (20 g) to produce NaAl(OH)_4_. Then PEG 4000 (0.3%) was added to solution and it was neutralized with HCl (0.1 M), to pH 8 until Al(OH)_3_ produced again.

The obtained precipitate filtered and washed with distilled water. The as-dried solid was calcined in the furnace at 800 °C for 3 hours through atmospheric air to produce nano-γ-Al_2_O_3_ powder.

### 2.3 Preparation of nano-γ-Al_2_O_3_/SbCl_5_

To a mixture of nano-γ-Al_2_O_3_ (1 g) and CH_2_Cl_2_ (10 ml), SbCl_5_ (0.5 ml) was added drop wise in the well ventilated hood. The resulting suspension was stirred for 1 hour at room temperature, filtered, washed with CH_2_Cl_2_, and dried at room temperature.

### 2.4 General procedure for the preparation of 2,3-dihydroperimidines

Naphthalene-1,8-diamine (1 mmol), aromatic aldehydes (1 mmol) and nano-γ-Al_2_O_3_/SbCl_5_ (0.1 g) were grounded in a mortar with a pestle for a few minutes to obtain a homogeneous mixture. After completed conversion as indicated by TLC, 10 ml of ethanol was added then the heterogeneous catalyst was filtered. By adding crushed ice to filtrate, the pure products were obtained as white solids.

## 3. Results and discussion

In continuation of our investigation on the utilization of solid acids in organic synthesis, we have synthesized nano-γ-Al_2_O_3_/SbCl_5_ as a new nano catalyst and studied its efficiency in the synthesis of 2,3-dihydroperimidines at room temperature under grinding conditions.

**Fig. 1:**
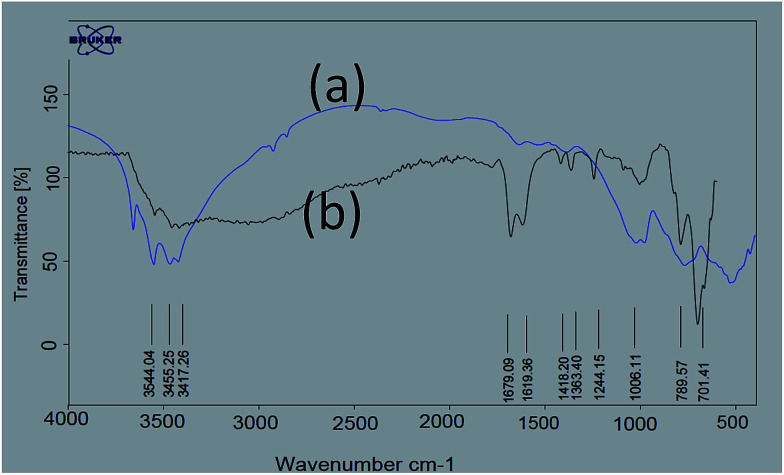
FT-IR spectra of (a) nano-γ-Al_2_O_3_ and (b) nano-γ-Al_2_O_3_/SbCl_5_.

For exploration of the structure of nano-γ-Al_2_O_3_/SbCl_5_, we have studied FT-IR spectra of nano-γ-Al_2_O_3_ and nano-γ-Al_2_O_3_/SbCl_5_ (Fig. 1). In nano-γ-Al_2_O_3_ FT-IR spectrum, the band in the region of 500–1000 cm^−1^ is attributed to the stretching vibrations of the (Al–O) bond in γ-Al_2_O_3_ (Fig. 1). In nano-γ-Al_2_O_3_/SbCl_5_ spectrum, in addition to γ-Al_2_O_3_ signal, two additional band at 701 show binding of SbCl_5_ to γ-Al_2_O_3_.

**Fig. 2:**
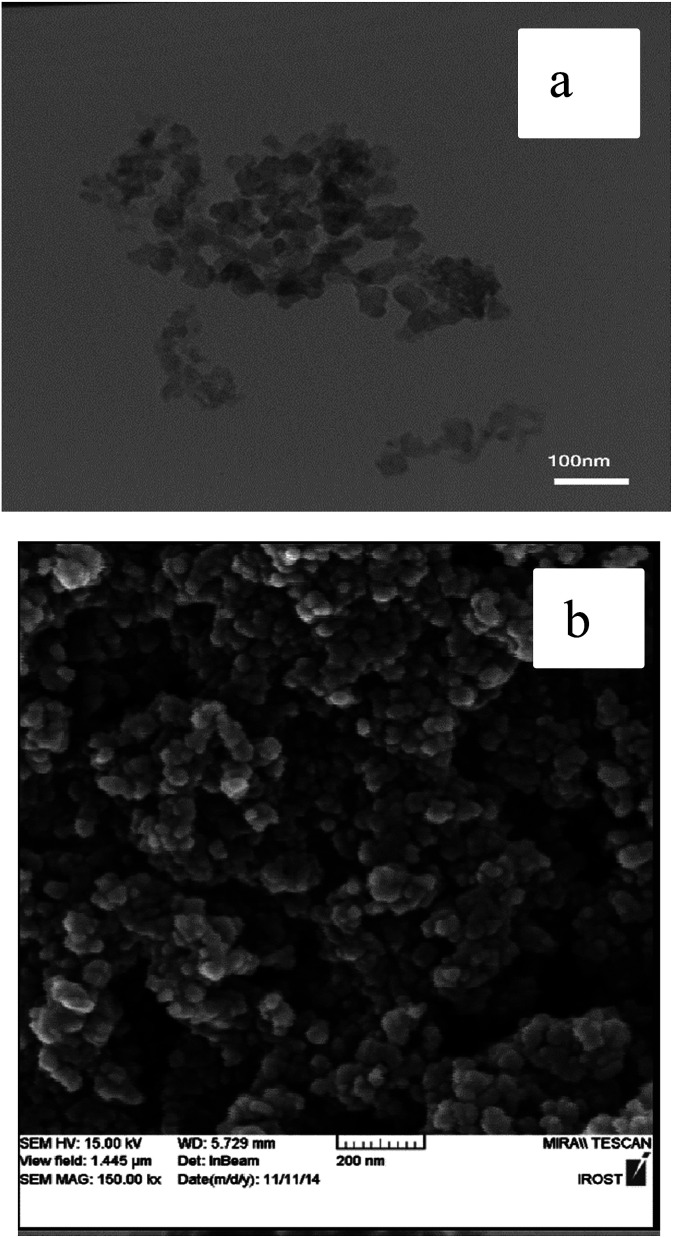
TEM (a) and FESEM (b) images of nano-γ-Al_2_O_3_/SbCl_5_.

The FESEM and TEM images of the nano-γ-Al_2_O_3_/SbCl_5_ are demonstrated in Fig. 2. They exhibit disordered spherical shape for nano particles below 50 nm.

**Fig. 3:**
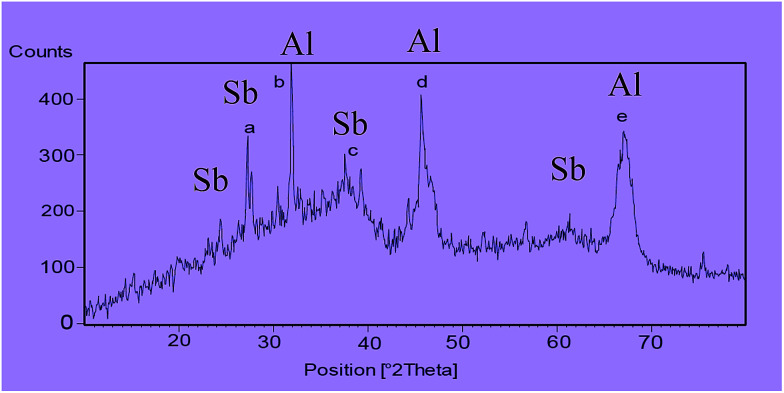
XRD patterns of nano-γ-Al_2_O_3_/SbCl_5_.

The X-ray diffraction (XRD) pattern of nano-γ-Al_2_O_3_/SbCl_5_ is exhibited in (Fig. 3). The signals at 2*θ* equal to 37 (c), 45 (d) and 67 (e) are displayed nano-γ-Al_2_O_3_ structure. According to XRD pattern, the two additional signals at 2*θ* equal to 28 (a) and 32 (b) respectively, are shown the presentment of bonded Sb to nano-γ-Al_2_O_3_ (Fig. 3).

**Fig. 4:**
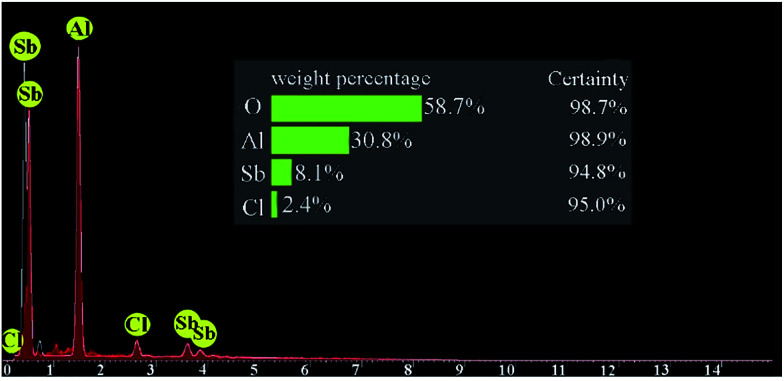
EDS analysis diagram of SbCl_5_/nano-γ-Al_2_O_3_.

The energy-dispersive X-ray spectroscopy (EDS) of the synthesized catalyst is displayed in Fig. 4. EDX pattern obviously approbates the presence of the anticipated elements in the construction of this catalyst and corroborated supporting of SbCl_5_ on nano-γ-Al_2_O_3_. The elemental compositions of nano-γ-Al_2_O_3_/SbCl_5_ were found to be 58.7, 30.8 and 8.1% for O, Al and Sb, respectively.

**Fig. 5:**
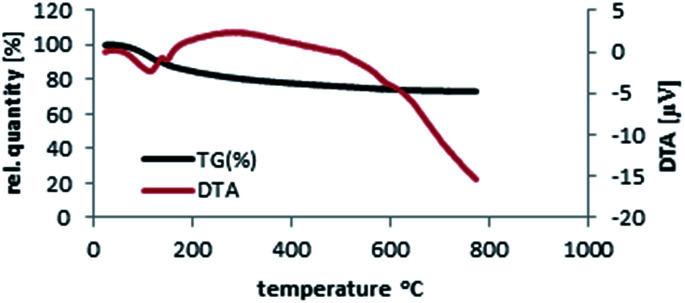
Thermal gravimetric analysis (TG-DTA) pattern of nano-γ-Al_2_O_3_/SbCl_5_.

Thermal gravimetric analysis (TG-DTA) template of SbCl_5_/nano-γ-Al_2_O_3_ was discovered by heating from 20 °C to 780 °C and then cooling until 165 °C (Fig. 5). The catalyst is stable until 390 °C and only 10.5% of its weight was reduce due to the removal of catalyst humidity. The char yield of the catalyst in 390 °C is 89.5%. According to the TG-DTA pattern of nano-γ-Al_2_O_3_/SbCl_5_ and our discussion, it was disclosed that this catalyst is appropriate for the advancement of organic reactions until 400 °C.

**Fig. 6:**
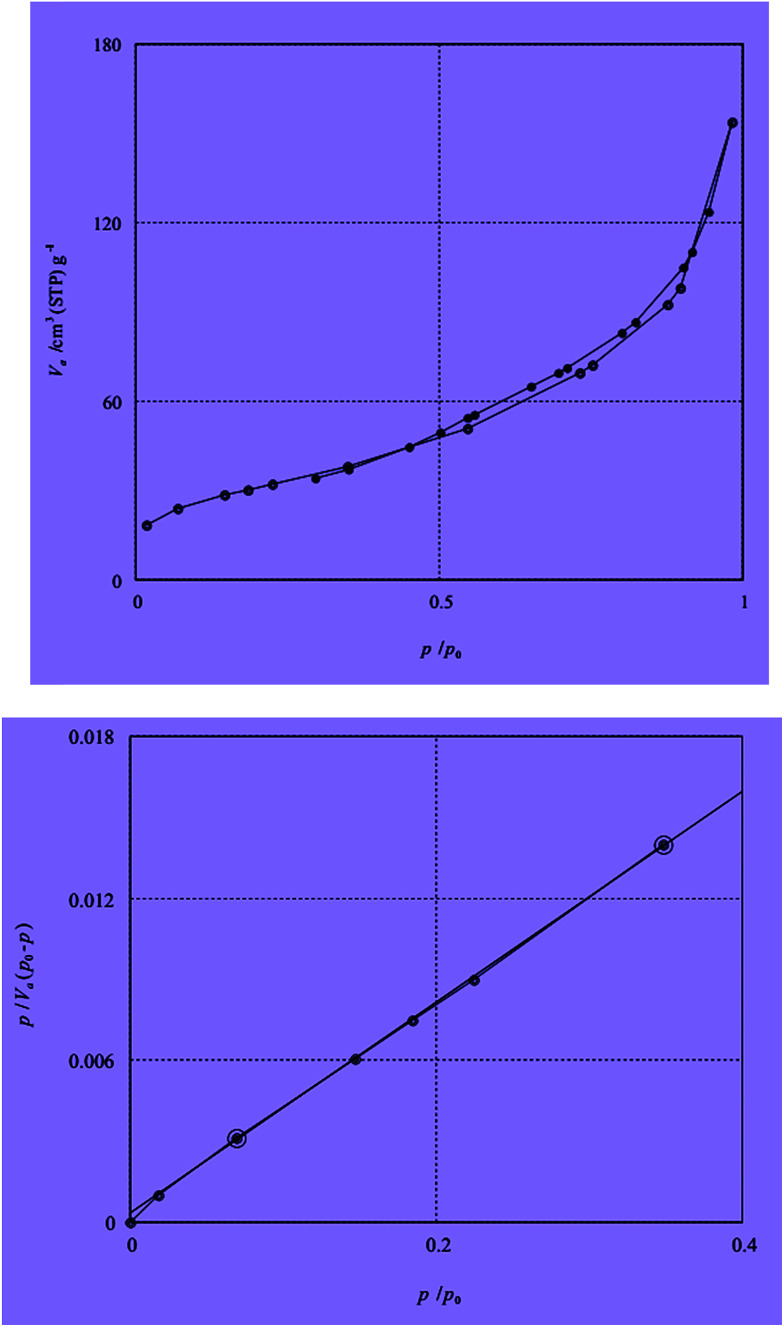
Nitrogen adsorption isotherm at 77 K of nano-γ-Al_2_O_3_/SbCl_5_.

The BET N_2_ adsorption method is applied to measure the surface area. The BET surface areas is assigned as 92.503 m^2^ g^−1^. The N_2_ adsorption isotherm of catalyst is described in Fig. 6. Inductive coupled plasma (ICP) analysis have determined the existence 200 mg of Sb in 1 g of catalyst.

After characterization of catalyst, we have investigated catalytic activity of nano-γ-Al_2_O_3_/SbCl_5_ for the synthesis of 2,3-dihydroperimidines derivatives. For optimization of the reaction reservations, 1,8-diaminonaphthalene (1 mmol), and 4-chlorobenzaldehyde (1 mmol) were used as model reactants under solvent-free conditions (Table 1). The best resultant based on yield and time of the reaction was afforded with 0.16 g of nano-γ-Al_2_O_3_/SbCl_5_. At first, in order to show the unrivalled catalytic behaviour of nano-γ-Al_2_O_3_/SbCl_5_ and to contrast its activity with other catalysts. Also, Table 1, shows the performance of our nano-catalyst in the preparation of 2,3-dihydroperimidines contrast to that of other reported methods.

**Table 1:** Condensation of 4-chlorobenzaldehyde and 1,8-diaminonaphthalene under various conditions^a^

Entry	Catalyst	Solvent	Temp (°C)	Time (min)	Yield%
1	Zeolite	Ethanol	r. t.	2700	40 (ref. 11)
2	Fe_3_O_4_/SiO_2_/(CH_2_)_3_N^+^Me_3_Br_3_^−^	—	80	15	95 (ref. 18)
3	FePO_4_	Ethanol	r. t.	420	90 (ref. 17)
4	Nano-γ-Al_2_O_3_/SbCl_5_ (0.005 g)	—	r. t.	60	20
5	Nano-γ-Al_2_O_3_/SbCl_5_ (0.008 g)	—	r. t.	60	30
6	Nano-γ-Al_2_O_3_/SbCl_5_ (0.01 g)	—	r. t.	60	35
7	Nano-γ-Al_2_O_3_/SbCl_5_ (0.08 g)	—	r. t.	30	50
8	Nano-γ-Al_2_O_3_/SbCl_5_ (0.1 g)	—	r. t.	15	70
9	Nano-γ-Al_2_O_3_/SbCl_5_ (0.14 g)	—	r. t.	15	80
**10**	**Nano-γ-Al**_**2**_ **O**_**3**_ **/SbCl**_**5**_ **(0.16 g)**	**—**	**r. t.**	**15**	**95**
11	Nano-γ-Al_2_O_3_/SbCl_5_ (0.20 g)	—	r. t.	15	95
12	Nano-γ-Al_2_O_3_/SbCl_5_ (0.25 g)	—	r. t.	15	95

a 1,8-Diaminonaphthalene (1 mmol), and 4-chlorobenzaldehyde (1 mmol) were used.

**Scheme 1:**
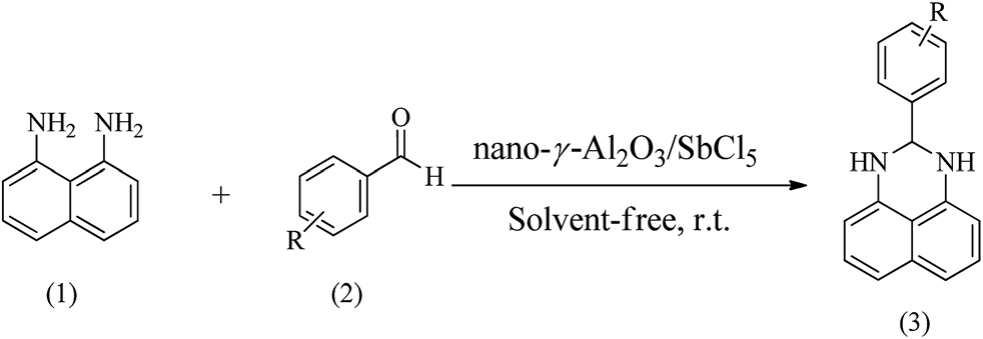
Synthesis of 2,3-dihydroperimidines.

Using the optimized reaction provisions, the reactions of various substituted benzaldehydes with naphthalene-1,8-diamine were studied (Scheme 1, Table 2).

**Table 2:** Synthesis of 2-substituted perimidines catalyst by nano-γ-Al_2_O_3_/SbCl_5_^a^

Entry	R	Product	Time (min)	Yield^b^ (%)
1	4-Cl	3a	14	95
2	2-NO_2_	3b	15	90
3	3-NO_2_	3c	13	95
4	4-NO_2_	3d	15	93
5	4-COOH	3e	20	80
6	4-NMe_2_	3f	20	90
7	4-OMe	3g	15	85
8	2,4-OMe_2_	3h	16	80
9	2,3-Cl_2_	3i	14	85
10	2,3-OMe_2_	3j	15	80
11	3,4-OMe_2_	3k	13	85

a 1,8-Diaminonaphthalene (1 mmol), aldehyde (1 mmol) and nano-γ-Al_2_O_3_/SbCl_5_ (0.16 g) were used.

b Isolated yield.

As displayed in Table 2, a number of aromatic aldehydes bearing electron withdrawing groups and electron-donating groups were further subjected to reaction employing a catalytic amount of nano-γ-Al_2_O_3_/SbCl_5_. In general, with electron-drawing substituents in the aromatic benzaldehydes, increased yields of products were generated, whereas the affect is reversed with electron donating substituents. However, the variations in the yields were little.

**Scheme 2:**
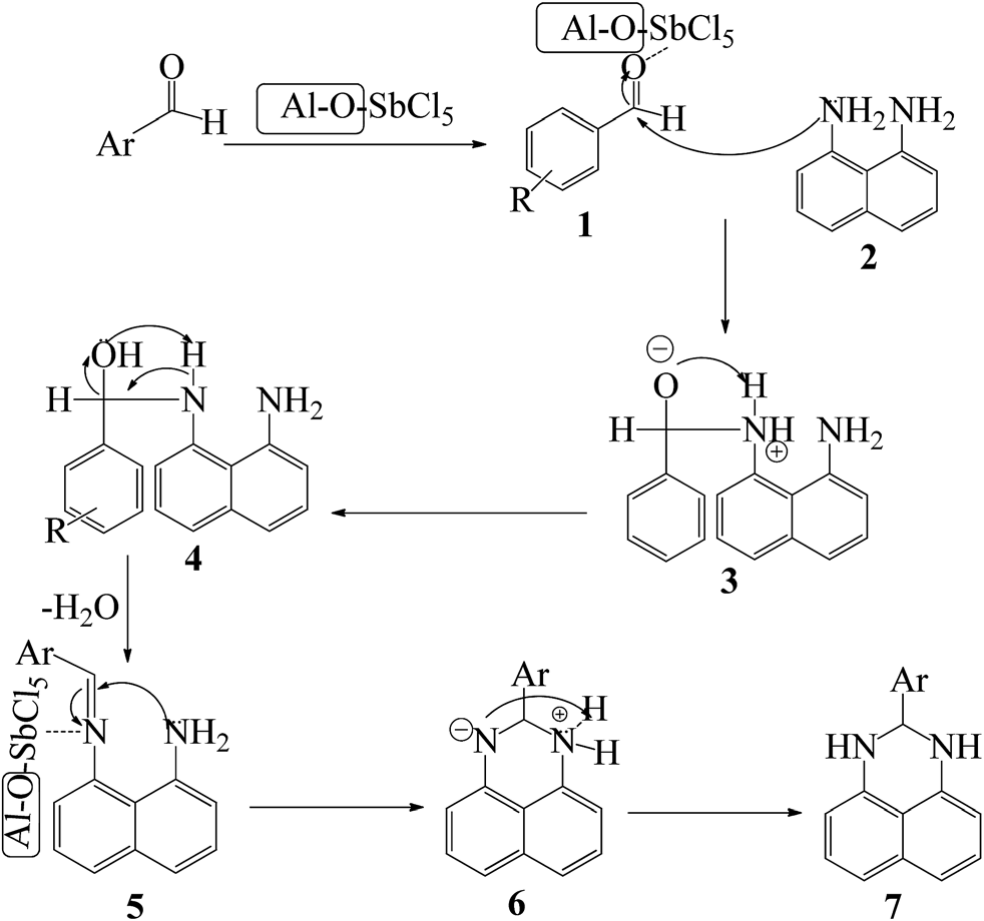
Proposed mechanism of the SbCl_5_/nano-γ-Al_2_O_3_-catalysed synthesis of 2,3-dihydroperimidines.

A plausible pathway for the preparation of 2,3-dihydroperimidines in the presence of nano-γ-Al_2_O_3_/SbCl_5_ is revealed in Scheme 2. Nucleophilic attack of 1,8-diamino naphthalene 2 to SbCl_5_-activated aldehyde 1 generated intermediate 3. *In situ* dehydration of compound 4 and nucleophilic attack of the second amino group to SbCl_5_-activated imine intermediate 5 afforded intermediate 6 to produce the compound 7.

## 4. Conclusions

In conclusion, nano-γ-Al_2_O_3_/SbCl_5_ was successfully synthesized, characterized and applied for the synthesis of 2,3-dihydroperimidine derivatives. Short reaction times, high conversions, clean reaction profiles, simple work-up, availability and high activity of catalyst, make this method suitable for many acid catalysed organic reactions.

## Conflicts of interest

There are no conflicts to declare.

## Supplementary Material

RA-008-C7RA13593A-s001
