# Supplementary material for: Nano-γ-Al2O3/SbCl5: an efficient catalyst for the synthesis of 2,3-dihydroperimidines
Source: RSC Adv. 2018 Feb 7;8(11):6178–82. doi: 10.1039/c7ra13593a (PMC9078262; doi:10.1039/c7ra13593a)
Supplement: RA-008-C7RA13593A-s001 [file RA-008-C7RA13593A-s001.pdf]

Electronic Supplementary Material (ESI) for RSC Advances.
This journal is © The Royal Society of Chemistry 2018

Nano-γ -Al_2O_3/$SbCl_5$: an efficient catalyst for the synthesis of 2, 3-dihydroperimidines

Abdolhamid Bamoniri*[a], Bi Bi Fatemeh Mirjalili[b] and Sepideh Saleh[a]

[a]*Departement of Org. Chem., Faculty of Chem., University of Kashan, Kashan, I. R. Iran*
[b]*Department of Chem., College of Sci., Yazd University, Yazd, I. R. Iran*

Physical and spectral data

2-(4-Chlorophenyl)-2, 3-dihydro-1*H*-perimidine (Table 2, 3a). IR (KBr): 3387, 3034, 2798, 1598, 1482, 1418 cm^{-1}. ^{1}HNMR (DMSO, 400MHz): δ 5.354 (s, 1H, CH), 6.47 (d, 2H, *J*=7.176). 6.790 (s, 1H, NH, exchange with D_2O), 6.97(d, 2H, *J*=7.66 Hz) 7.13 (d, 2H, *J*=7.55 Hz) 7.162 (s, 1H, NH, exchange with D_2O) 7.475 (d, 2H, *J*= 7.95Hz) 7.60 (d, 2H, *J*= 7.95Hz) ^{13}CNMR (DMSO) [28]: 66.4, 104.4,112.5, 115.3, 126.8, 127.9, 128.2, 128.5, 134.4, 141.8, 143.0.

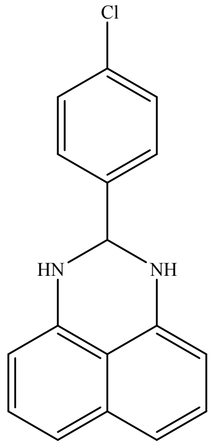

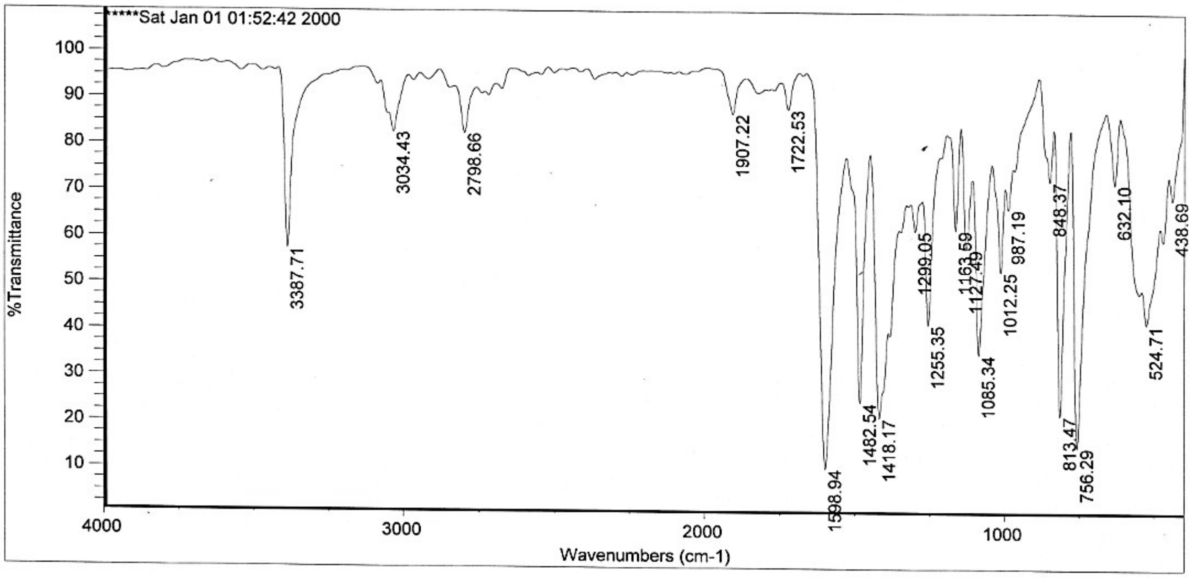

FT-IR spectrum of 2-(4-chlorophenyl)-2, 3-dihydro-1*H*-perimidine

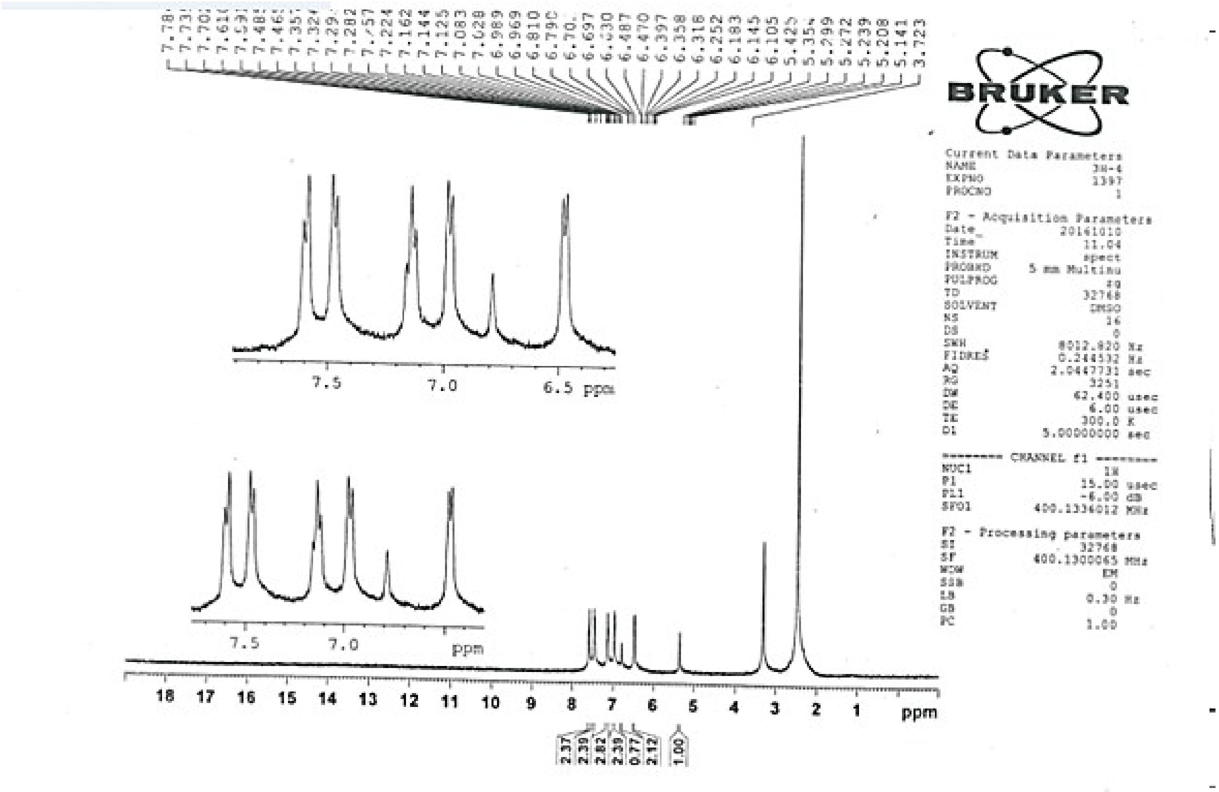

^{1}H NMR spectrum of 2-(4-chlorophenyl)-2, 3-dihydro-1*H*-perimidine

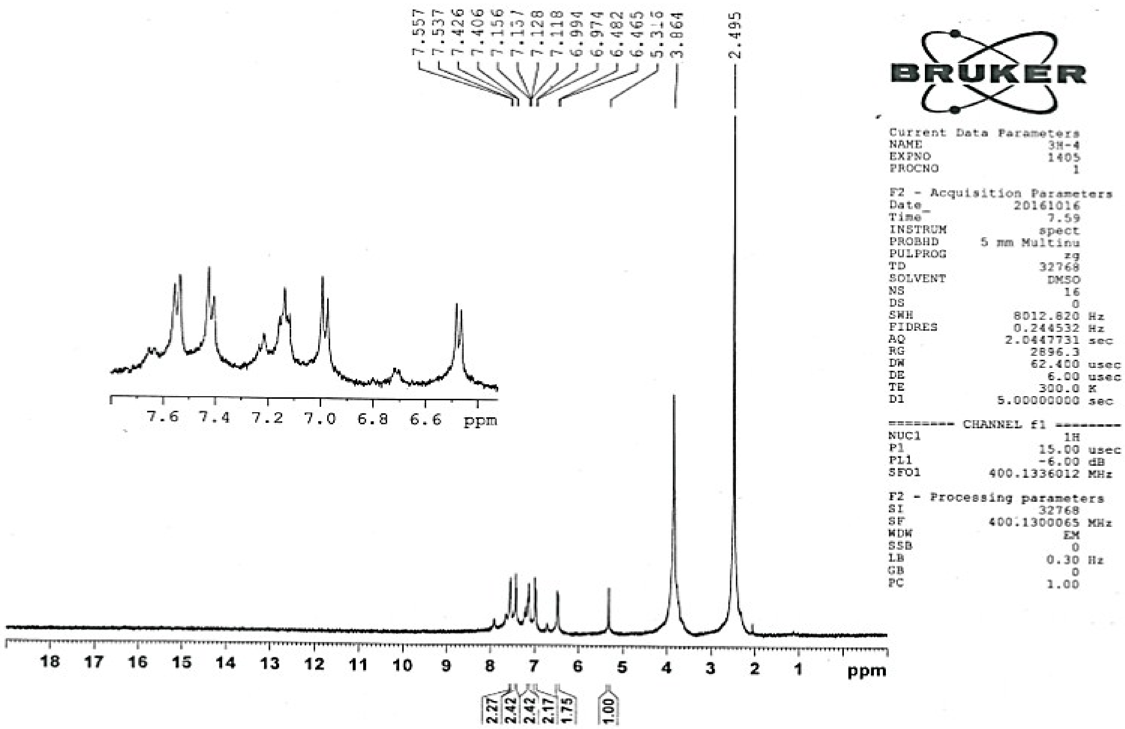

^{1}H NMR spectrum of 2-(4-chlorophenyl)-2, 3-dihydro-1*H*-perimidine in the presence of D_2O

2-(2-Nitroophenyl)-2, 3-dihydro-1*H*perimidine (Table 2, 3b). IR (KBr): 3359, 3230, 3046, 2848, 1603, 1517, 1412 cm^{-1}. ^{1}HNMR (DMSO, 400MHz): δ 3.345 (s, 1H, NH, exchange with D_2O), 5.792 (s, 1H, CH), 6.51 (d, 2H, *J*=7.2). 6.8 (s, 1H, NH, exchange with D_2O), 7.01 (d, 2H, *J*=8.4 Hz) 7.16(t, 2H, *J*=8 Hz) 7.61 (t, 2H, *J*=8 Hz) 7.75(t, 1H, *J*=6,8 Hz)7.89 (d, 2H, *J*= 8Hz) 8.02 (d, 2H, *J*= 8Hz) ^{13}CNMR (DMSO, 75 MHz) [23]: δ 61.4, 105.2, 112.5, 116.2, 124.6 127.5, 130.0, 130.4, 134.0, 134.7, 136.9, 142.5, 149.4.

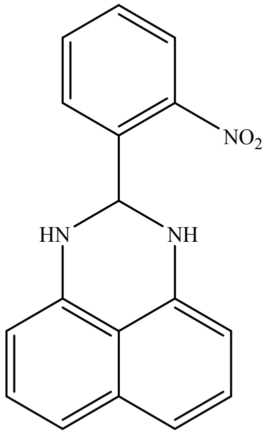

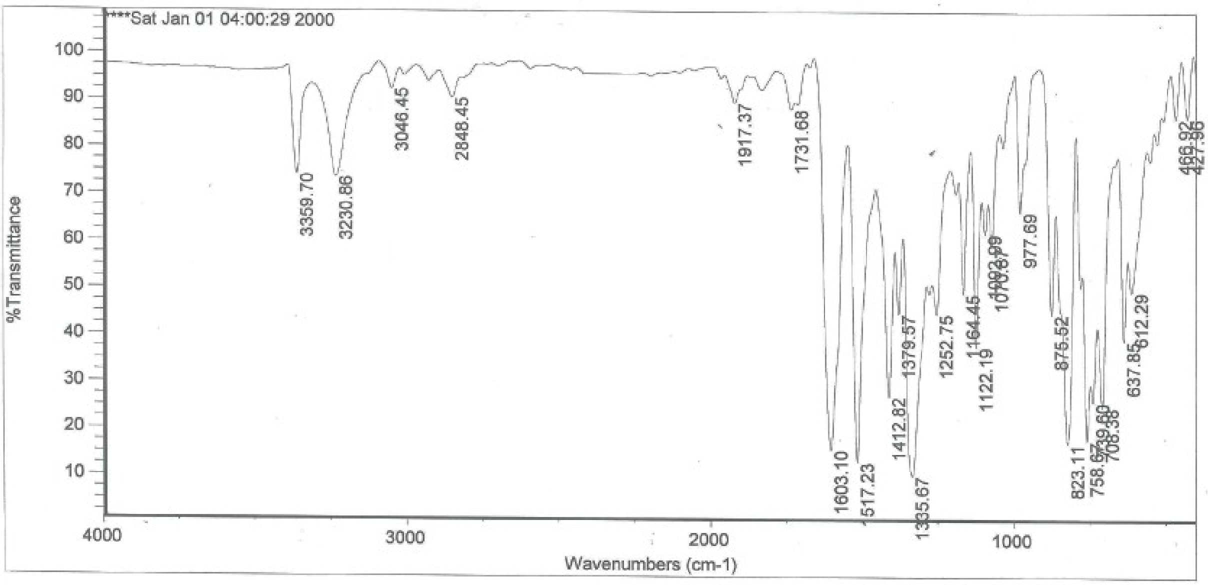

FT-IR spectrum of 2-(2-nitroophenyl)-2, 3-dihydro-1*H*perimidine

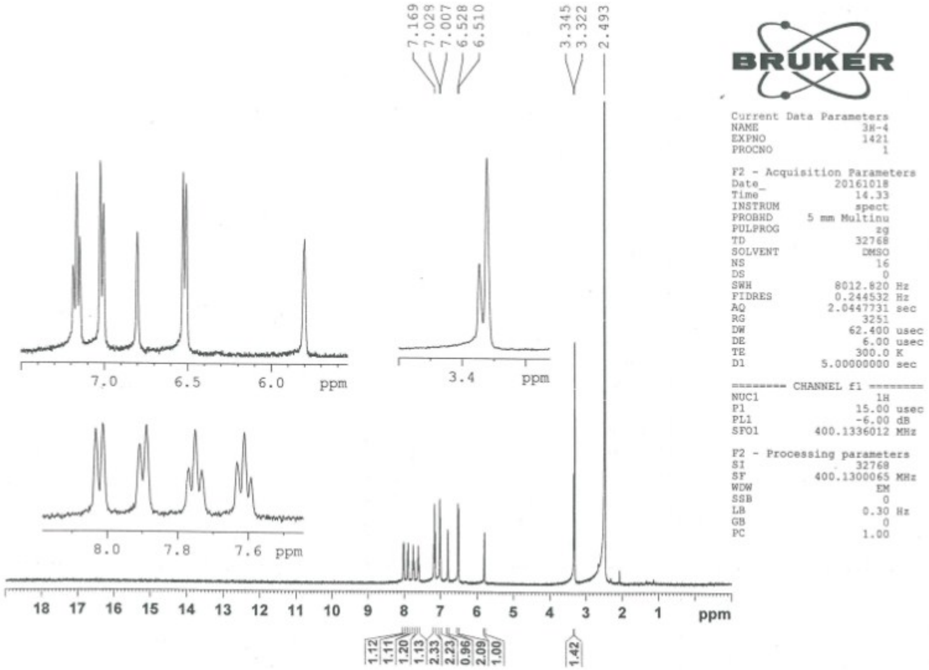

^{1}H NMR spectrum of 2-(2-nitroophenyl)-2, 3-dihydro-1*H*perimidine

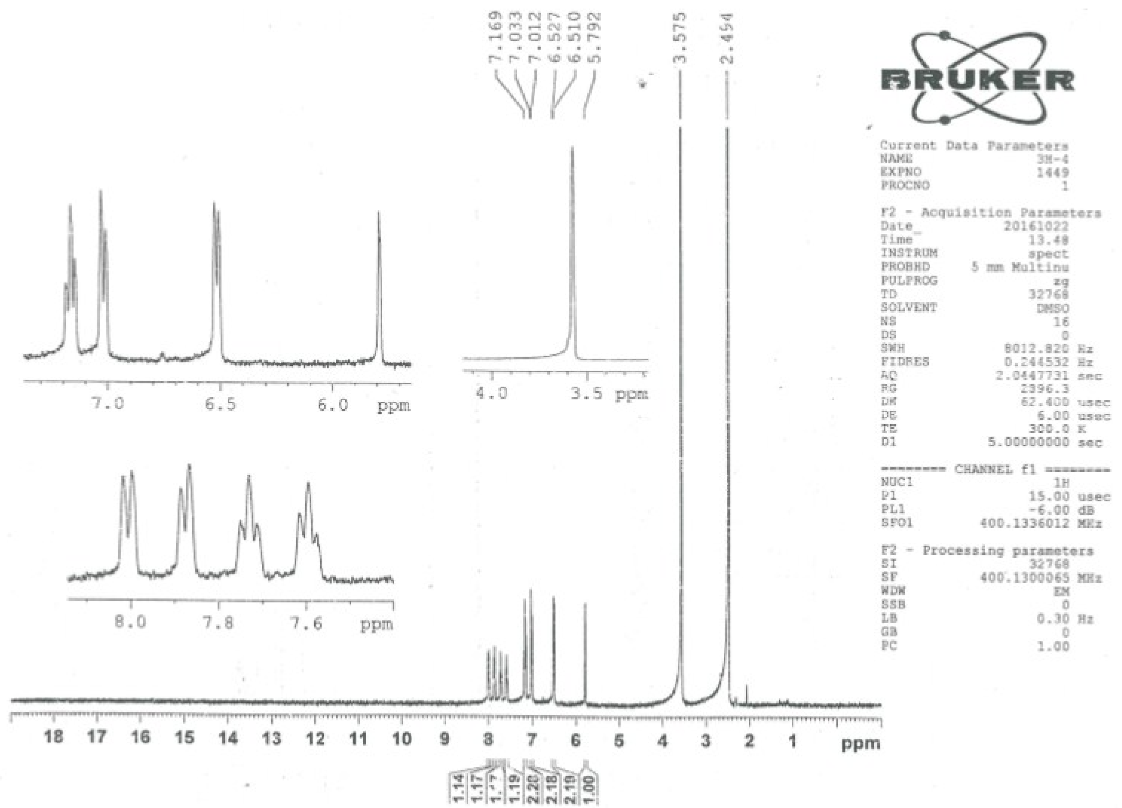

^1H NMR spectrum of 2-(2-nitroophenyl)-2, 3-dihydro-1*H*perimidine in the presence of D_2O

2-(3-Nitroophenyl)-2, 3-dihydro-1*H*perimidine (Table 2, 3c). IR (KBr): 3343, 3227, 3039, 2925, 1602, 1526, 1416 cm^{-1}. ^{1}HNMR (DMSO, 400MHz): 5.6 (s, 1H, CH), 6.50 (d, 2H, *J*=7.2 Hz). 6.976 (s, 1H, NH, exchange with D_2O), 7 (d, 2H, *J*=8.4 Hz) 7.158(t, 2H, *J*=7.6 Hz) 7.66 (t, 1H, *J*=8 Hz) 7.98(d, 1H, *J*= 7.6 Hz), 8.19 (d, 1H, *J*= 6.8Hz) 8.37 (s, 1H) ^{13}CNMR (DMSO, 75 MHz) [23]: δ 65.3, 105.1, 112.9, 116.1, 123.1, 123.7, 127.4, 130.4, 134.8, 135.1, 142.7, 145.0, 148.1.

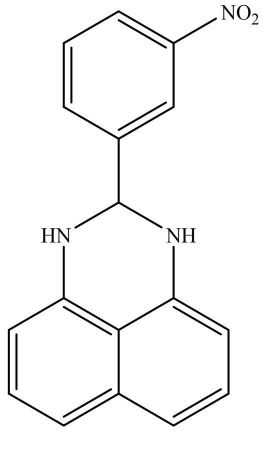

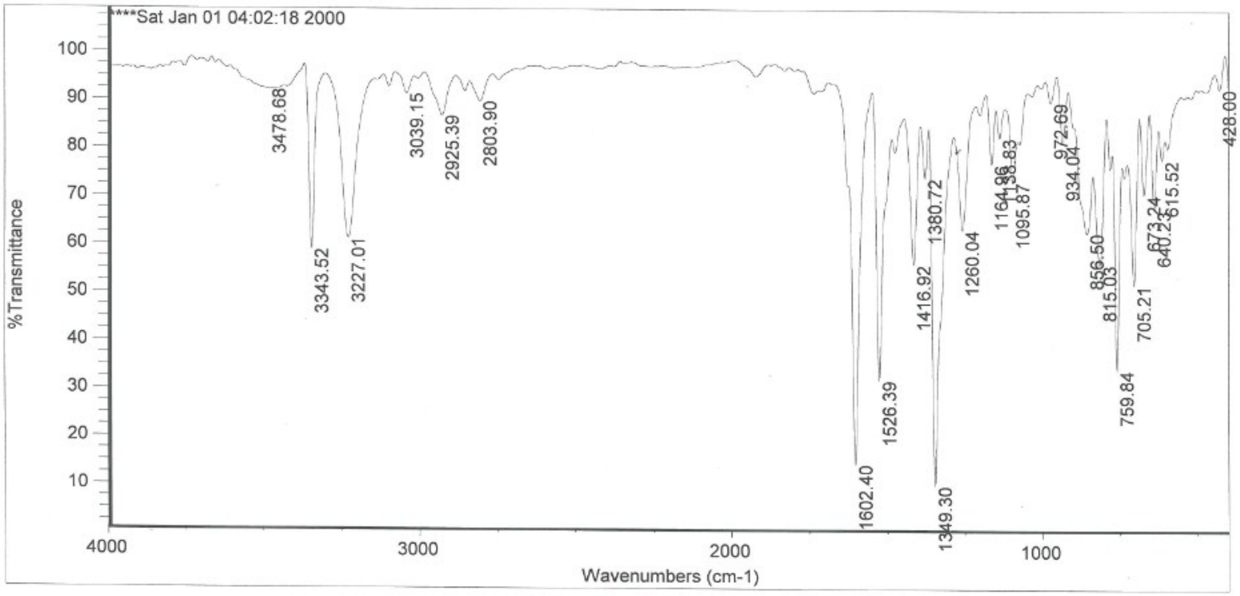

FT-IR spectrum 2-(3-Nitroophenyl)-2, 3-dihydro-1*H*perimidine

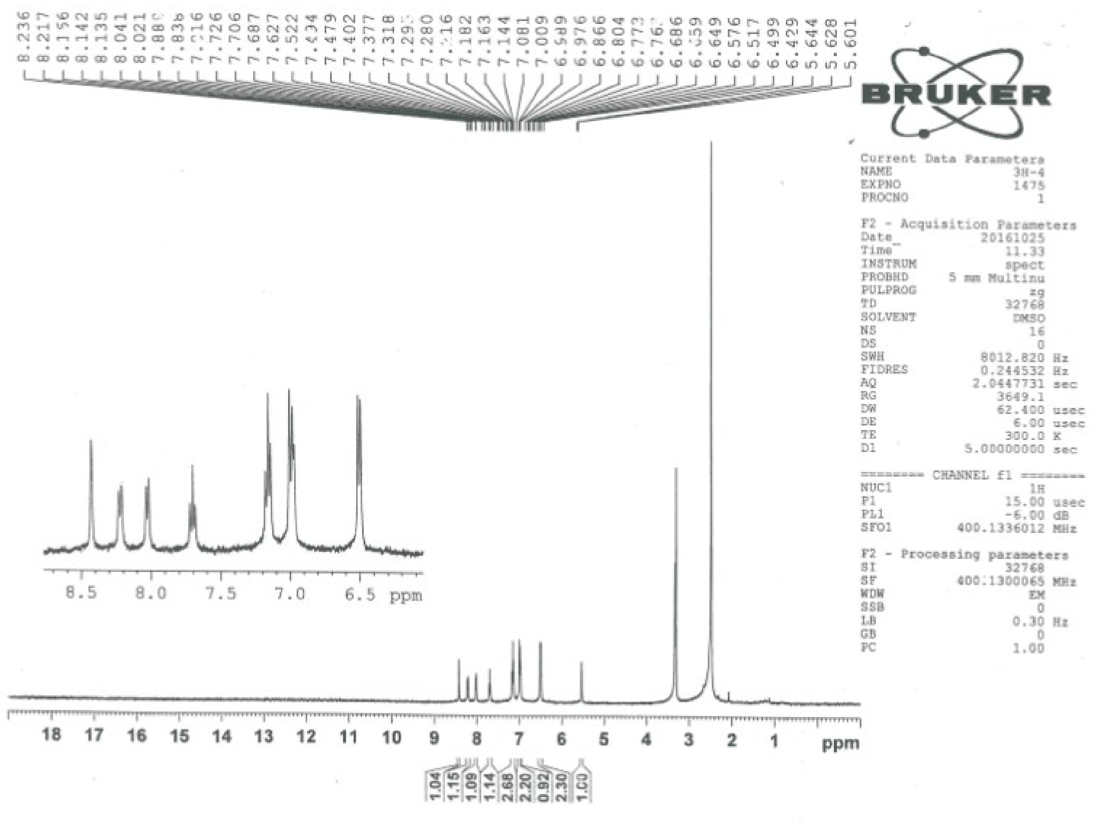

^{1}H NMR spectrum 2-(3-Nitroophenyl)-2, 3-dihydro-1*H*perimidine

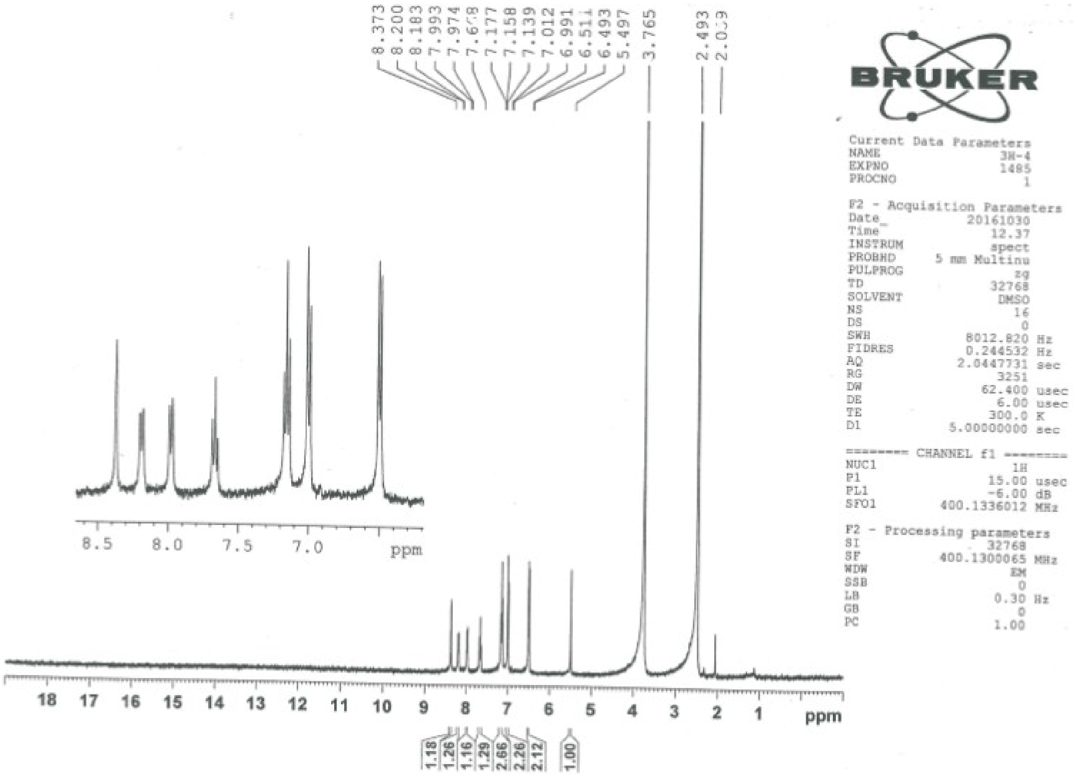

^{1}H NMR spectrum 2-(3-Nitroophenyl)-2, 3-dihydro-1*H*perimidine in the presence of D_2O

2-(4-Nitroophenyl)-2, 3-dihydro-1*H*perimidine (Table 2, 3d). IR (KBr): 3365, 3100, 3066, 2798, 1601, 1512, 1413 cm^{-1}. ^{1}HNMR (DMSO, 400MHz): 3.34 (s, 1H, NH, exchange with D_2O), 5.52 (s, 1H, CH), 6.49 (d, 2H, *J*=6.8 Hz). 6.98 (d, 3H, *J*=8.8 Hz), 7.158(t, 2H, *J*=7.6 Hz), 8.19 (d, 2H, *J*= 8.8Hz) 8.25 (d, 2H, J= 8.4Hz) ^{13}CNMR (DMSO) [28]: δ 64.8, 104.5, 112.3, 115.5, 123.3, 126.8, 128.9, 134.2, 141.9, 147.3, 149.8.

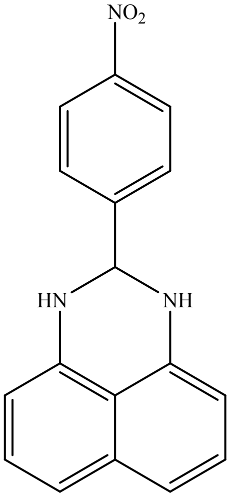

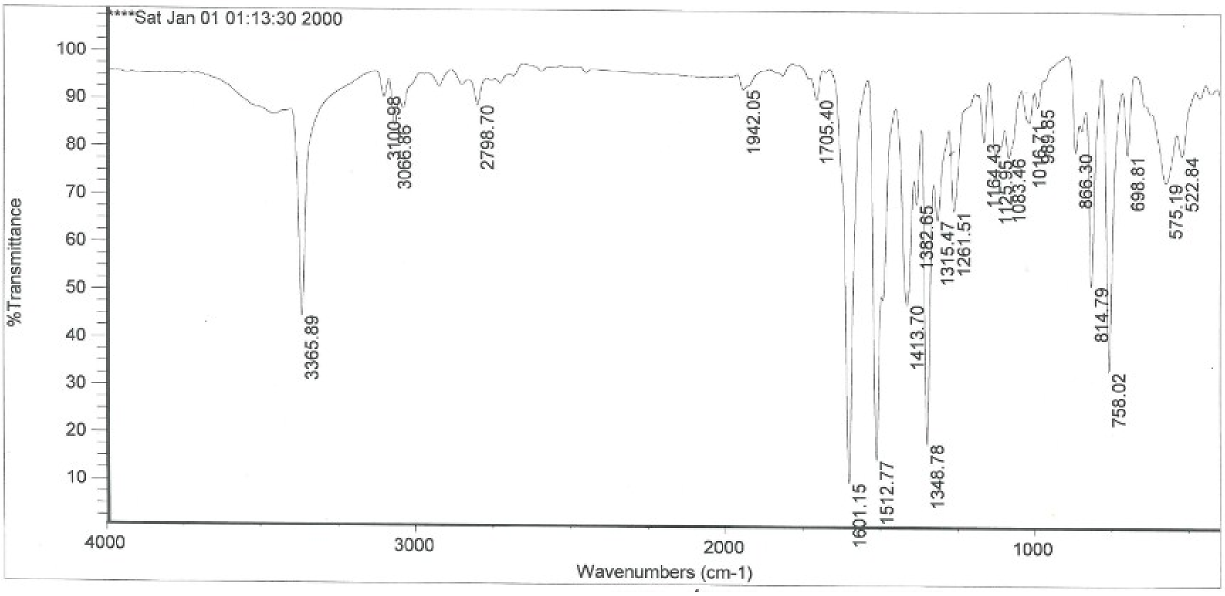

FT-IR spectrum of 2-(4-Nitroophenyl)-2, 3-dihydro-1*H*perimidine

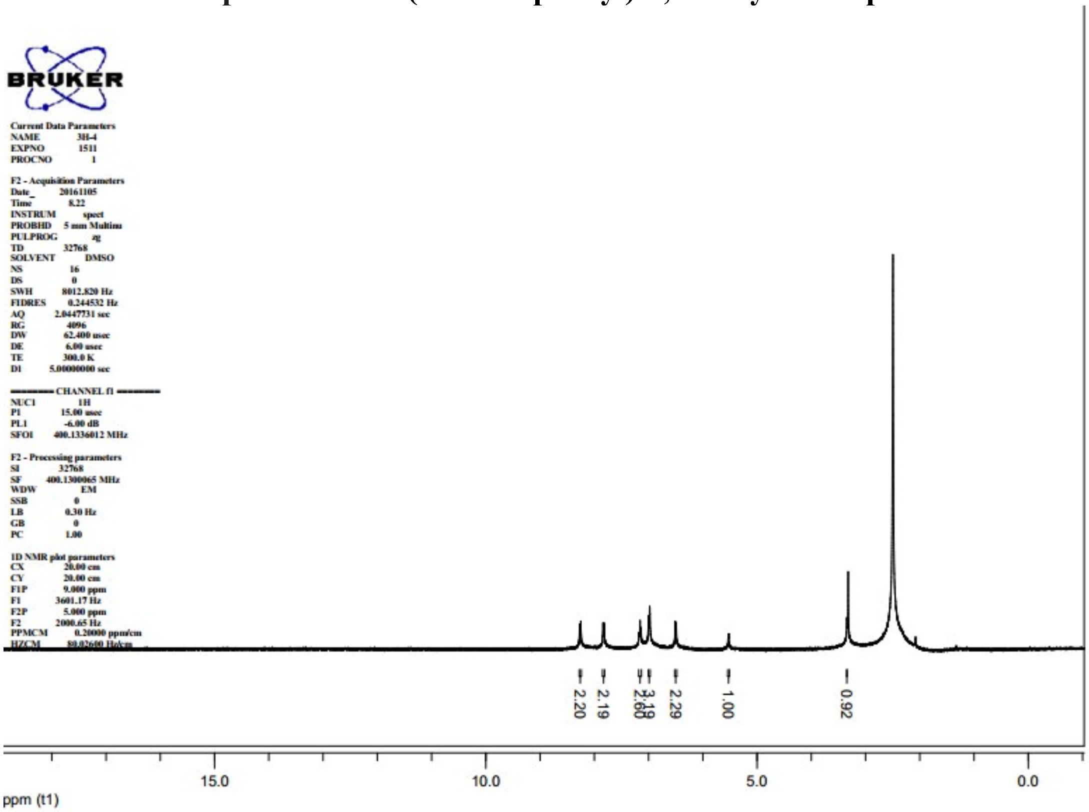

1H NMR spectrum of 2-(4-Nitroophenyl)-2, 3-dihydro-1*H*perimidine

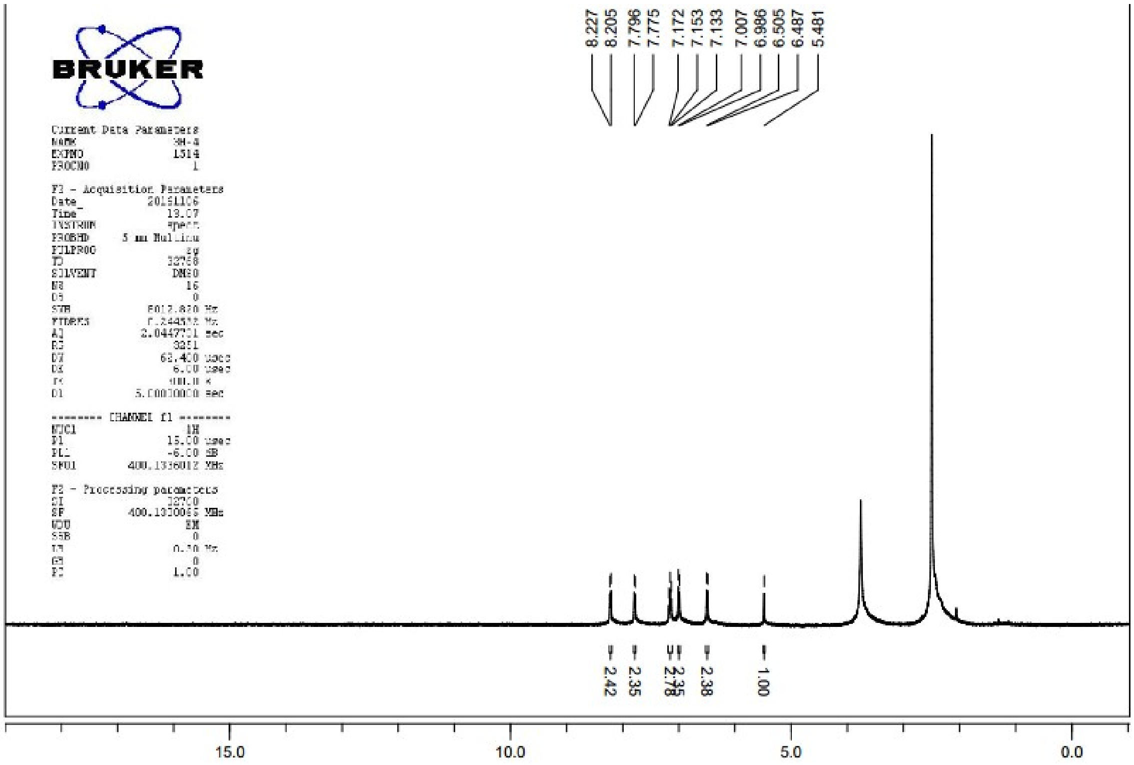

^{1}H NMR spectrum of 2-(4-Nitroophenyl)-2, 3-dihydro-1*H*perimidine in the presence of D_2O

2-(4-Carboxyphenyl)-2, 3-dihydro-1*H*perimidine (Table 2, 3e). IR (KBr): 3622, 3374, 3230, 2924, 1685, 1599, 1480, 1413 cm^{-1}. ^{1}HNMR (DMSO, 400MHz): 5.42 (s, 1H, CH), 6.49 (d, 2H, *J*=7.2 Hz), 6.85 (s, 2H, NH, exchange with D_2O), 6.98 (d, 2H, *J*=8 Hz), 7.146 (t, 2H, *J*=7.6 Hz), 7.69 (d, 2H, J= 8Hz), 7.97 (d, 2H, J= 8Hz). ^{13}CNMR (DMSO, 75 MHz): ?

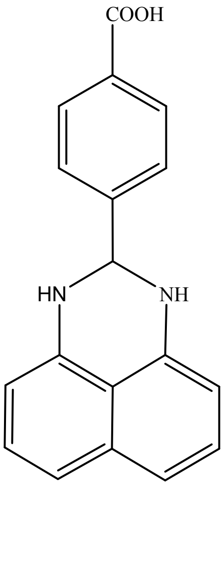

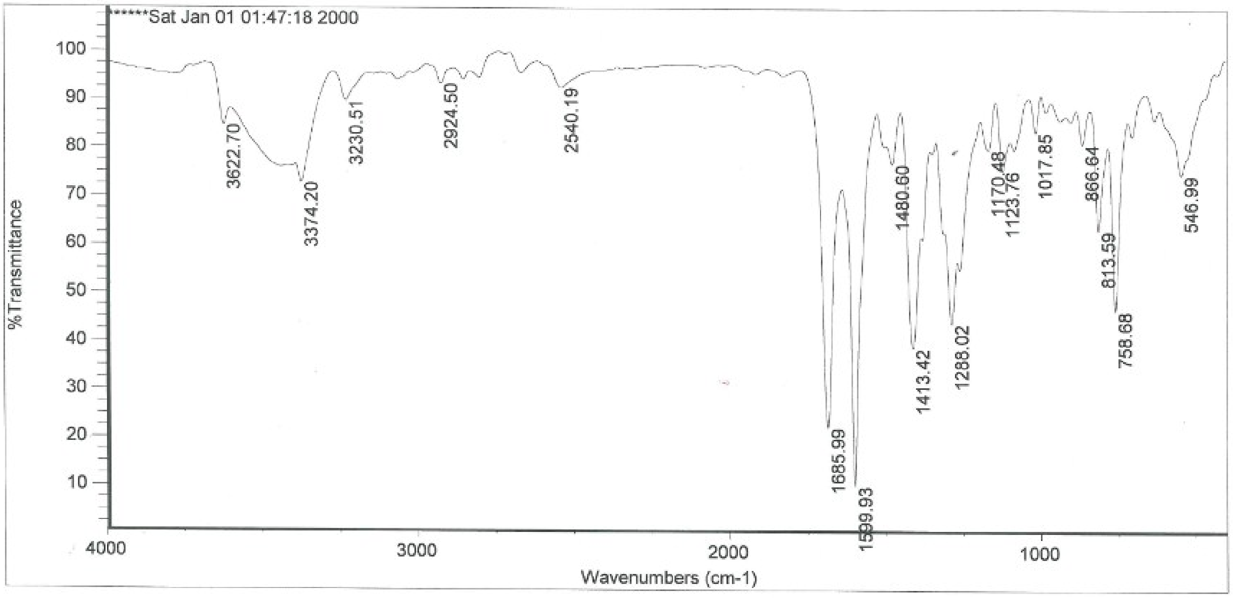

FT-IR spectrum of 2-(4-Carboxyphenyl)-2, 3-dihydro-1*H*perimidine

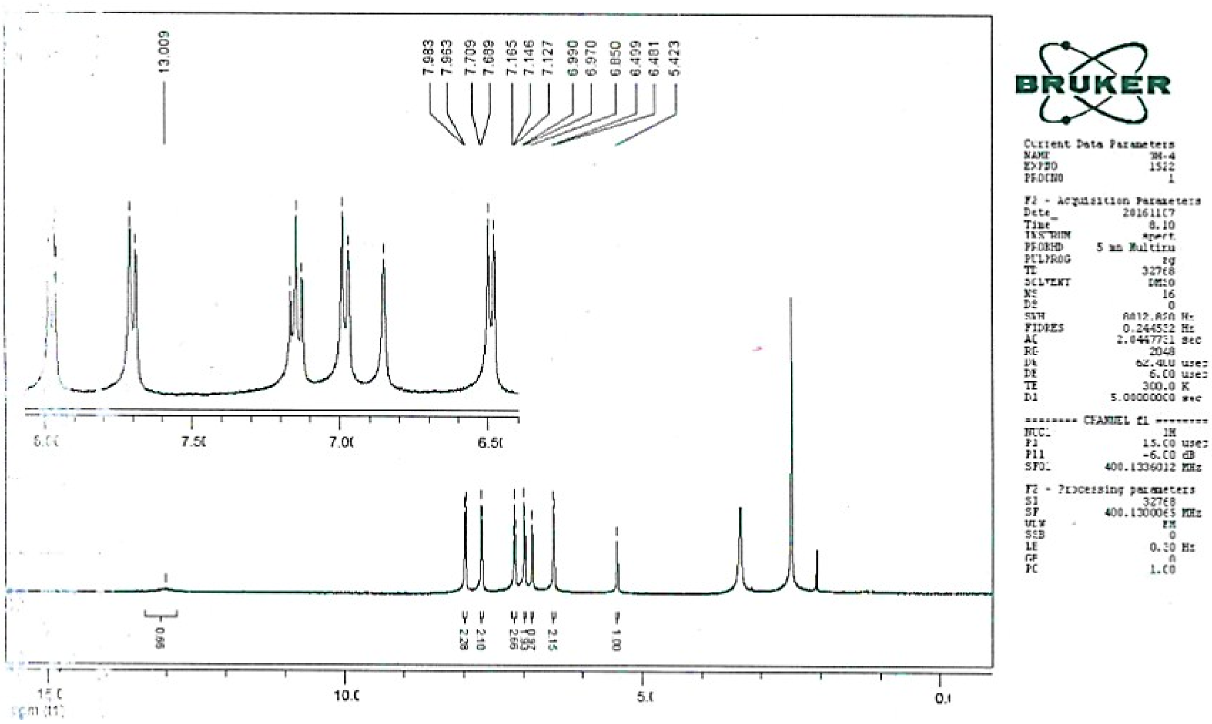

^{1}H NMR spectrum of 2-(4-Carboxyphenyl)-2, 3-dihydro-1*H*perimidine

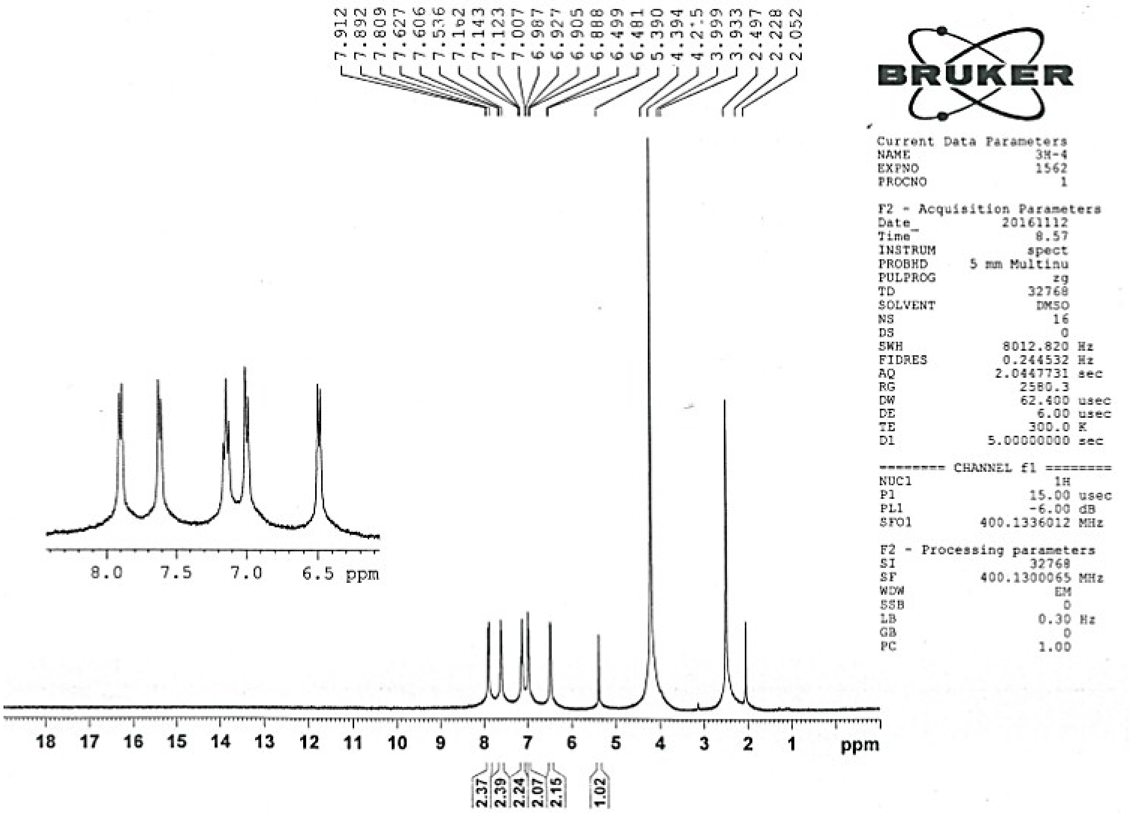

H NMR spectrum of 2-(4-Carboxyphenyl)-2, 3-dihydro-1*H*perimidine in the presence of D_2O

2-(4-Dimethylaminophenyl)-2, 3-dihydro-1*H*perimidine (Table 2, 3f). IR (KBr): 3367, 3038, 2891, 1598, 1527, 1478, 1414 cm^{-1}. ^{1}HNMR (DMSO, 400MHz): 2.891 (s, 6H, $2CH_3$), 3.34(s, 1H, NH, exchange with D_2O), 5.21 (s, 1H, CH), 6.45 (d, 2H, *J*=7.2 Hz), 6.57 (s, 1H, NH, exchange with D_2O), 6.75 (d, 2H, *J*=8 Hz), 6.94 (d, 2H, *J*=8 Hz), 7.11 (t, 2H, *J*=6.8 Hz), 7.39 (d, 2H, *J*=7.2 Hz), ^{13}CNMR (DMSO) [28]: 40.2, 66.2, 104.1, 111.9, 112.4, 114.9, 126.7, 128.4, 129.0, 134.3, 143.4, 150.8.

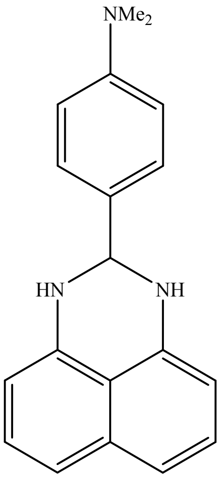

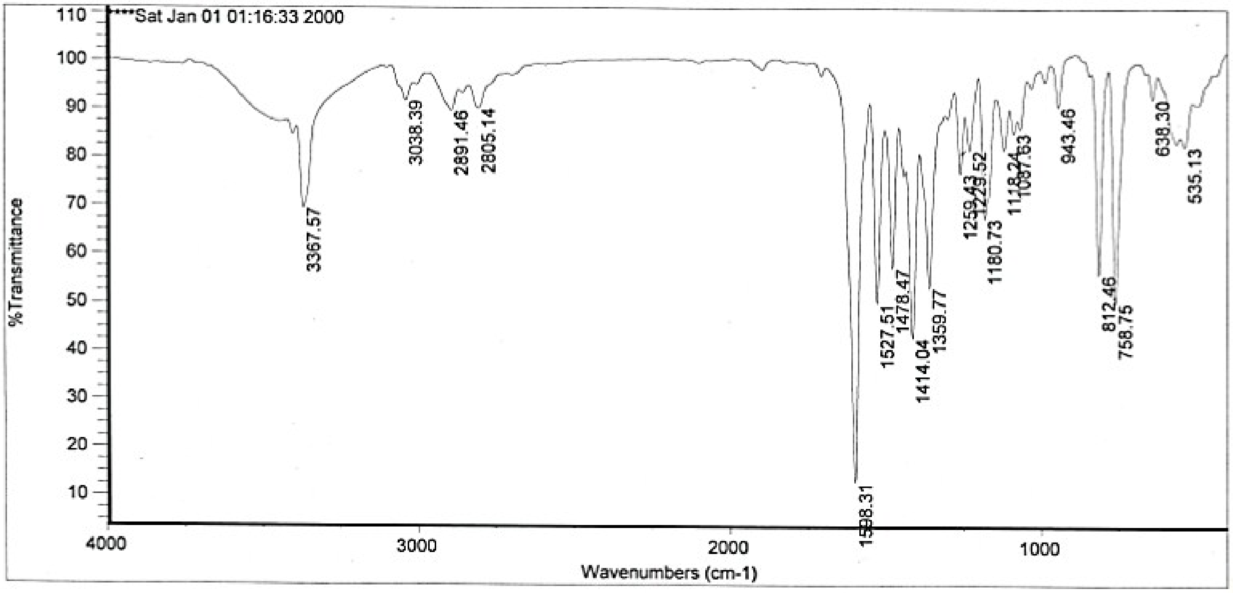

FT-IR spectrum of 2-(4-Dimethylaminophenyl)-2, 3-dihydro-1*H*perimidine

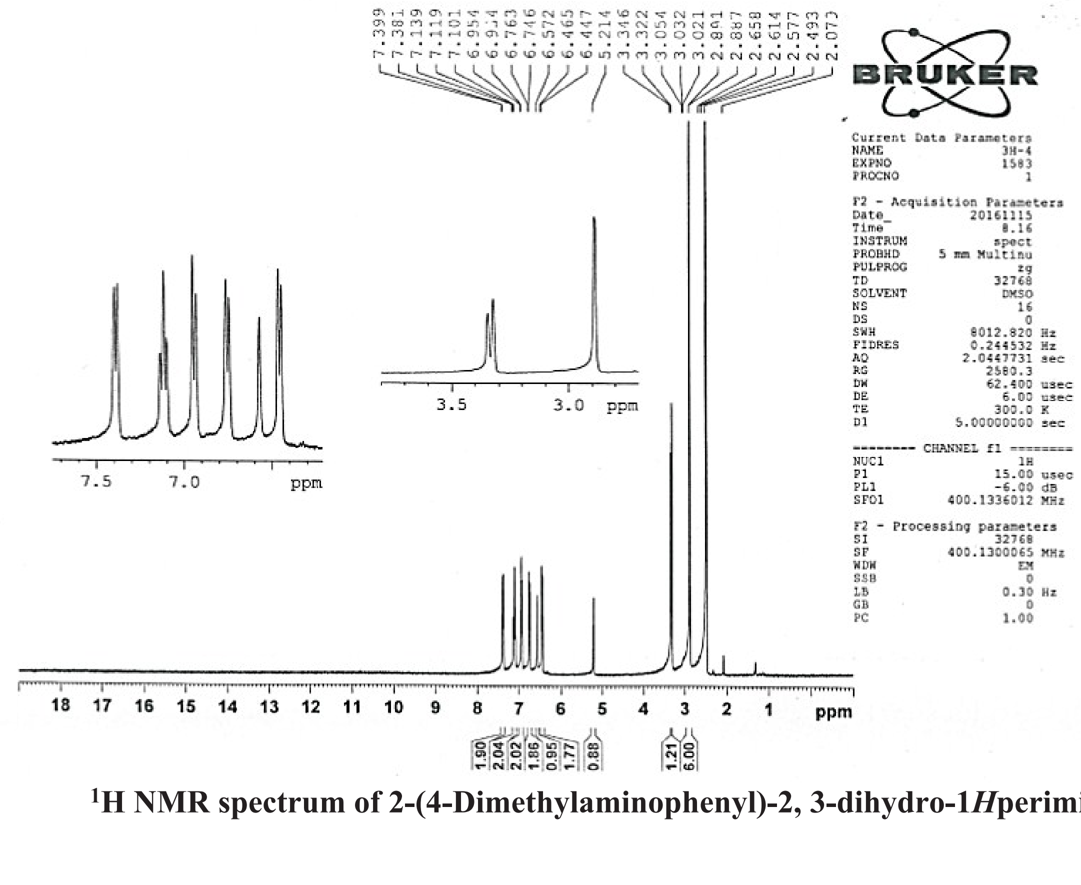

^{1}H NMR spectrum of 2-(4-Dimethylaminophenyl)-2, 3-dihydro-1*H*perimidine

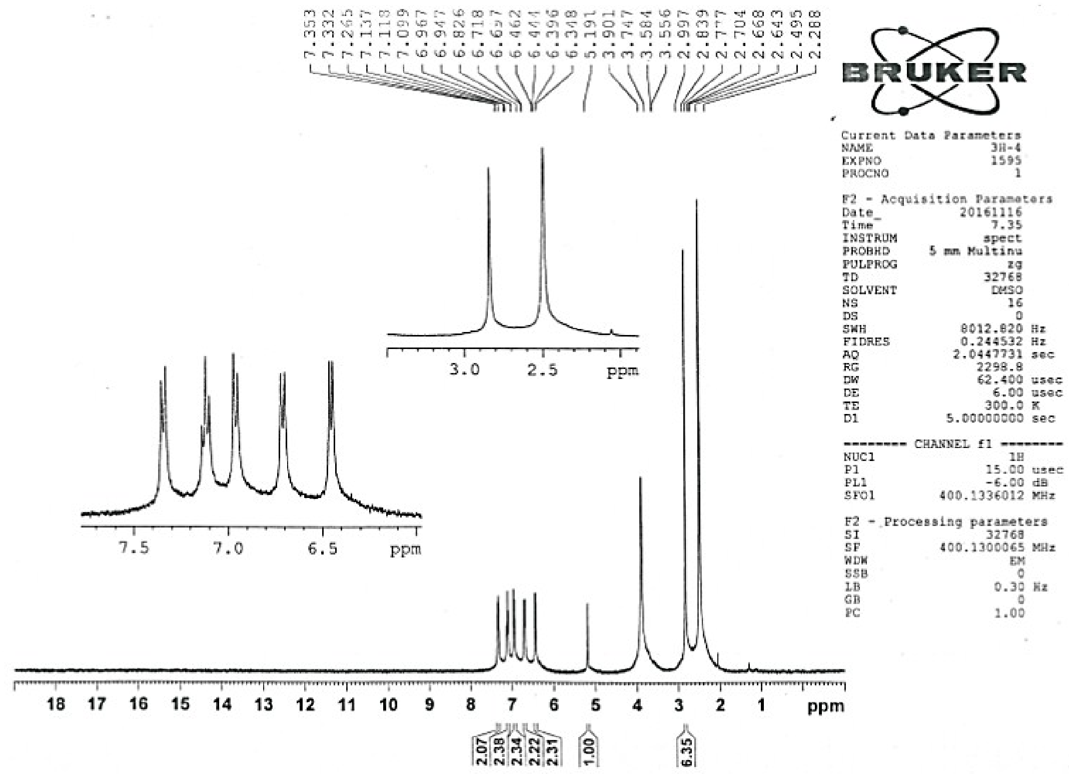

^{1}H NMR spectrum of 2-(4-Dimethylaminophenyl)-2, 3-dihydro-1*H*perimidine in the presence of D_2O

2-(4-methoxyphenyl)-2, 3-dihydro-1*H*perimidine (Table 2, 3g). IR (KBr): 3365, 3036, 2922, 1597, 1511, 1484, 1408 cm^{-1}. ^{1}HNMR (DMSO, 400MHz): 3.76 (s, 3H, CH_3), 5.28 (s, 1H, CH), 6.46 (d, 2H, *J*=7.2 Hz), 6.56 (s, 2H, NH, exchange with D_2O), 6.96 (m, 4H, *J*=6.8 Hz), 7.12 (t, 2H, *J*=8 Hz), 7.50 (d, 2H, *J*=7.2 Hz), ^{13}C NMR (DMSO) [28]: 55.2, 66.0, 104.3, 112.5, 113.5, 115.2, 126.8, 129.1, 133.8, 134.4, 143.2, 159.5.

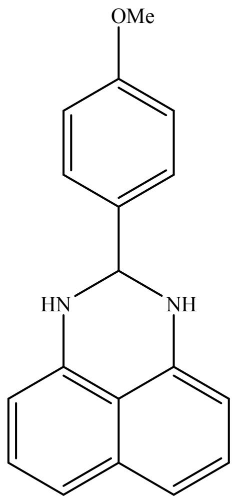

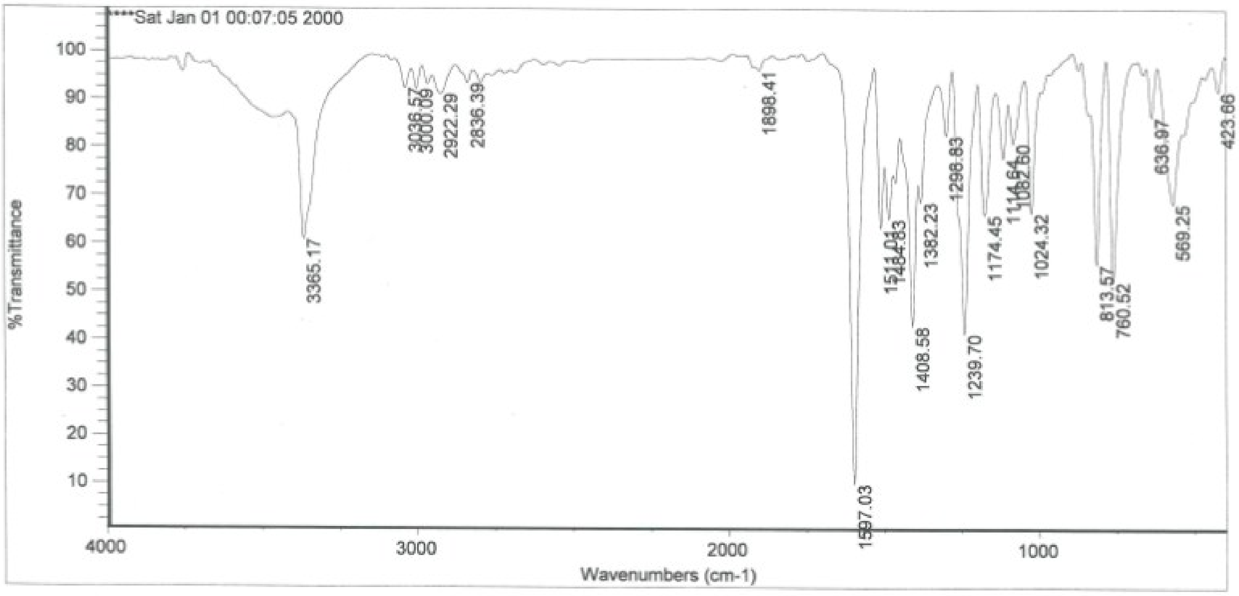

FT-IR spectrum of 2-(4-methoxyphenyl)-2, 3-dihydro-1*H*perimidine

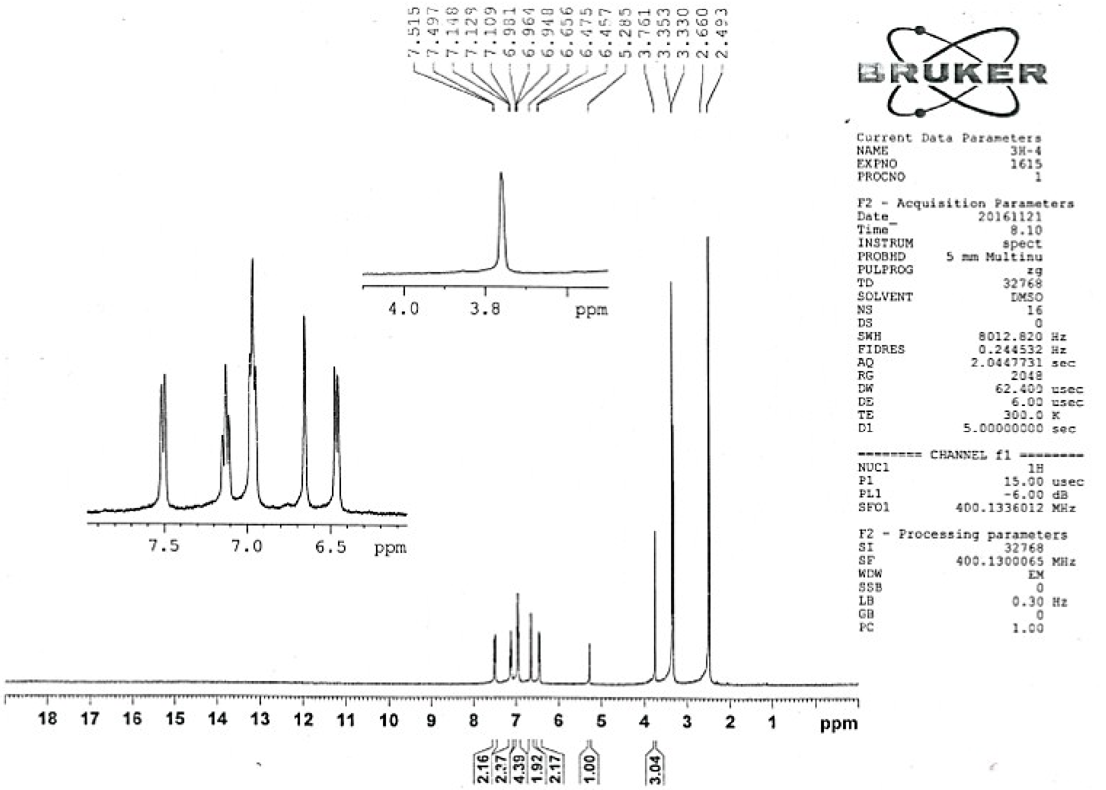

^{1}H-NMR spectrum of 2-(4-methoxyphenyl)-2, 3-dihydro-1*H*perimidine

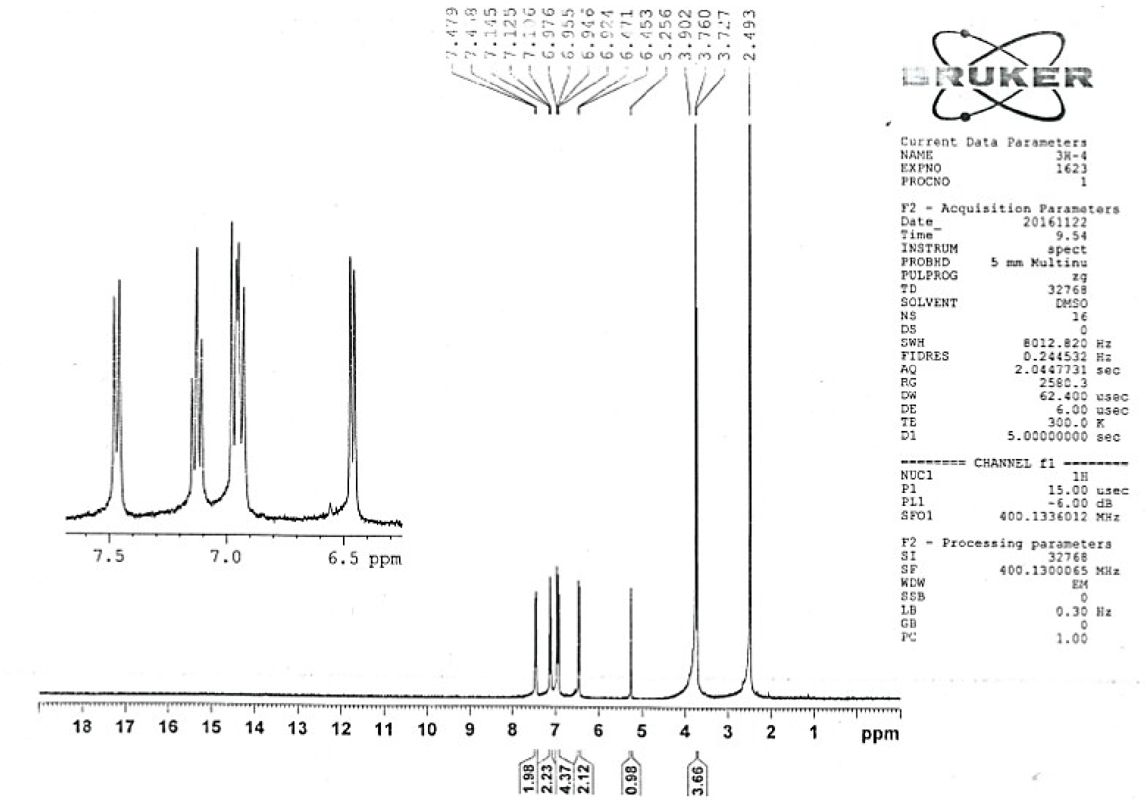

^{1}H NMR spectrum of 2-(4-methoxyphenyl)-2, 3-dihydro-1*H*perimidine in the presence of D_2O

2-(2, 4-Dimethoxyphenyl)-2, 3-dihydro-1*H*perimidine (Table 2, 3h). IR (KBr): 3378, 3043, 2959, 1600, 1499, 1460, 1415 cm^{-1}.^{1}HNMR (DMSO, 400MHz): 3,76 (s, 3H, CH_3), 3.81 (s, 3H, CH_3), 5.60 (s, 1H, CH), 6.44 (s, 1H, NH, exchange with D_2O), 6.47 (d, 2H, *J*=7.2 Hz), 6.56 (d, 1H, *J*=8 Hz), 6.60 (s, 1H), 6.94 (d, 2H, *J*= 7.6Hz), 7.12 (t, 2H, *J*= 8Hz), 7.44 (d, 1H, *J*= 8Hz) ^{13}C NMR (DMSO): ?

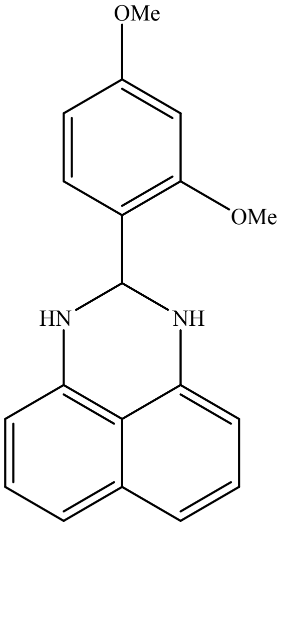

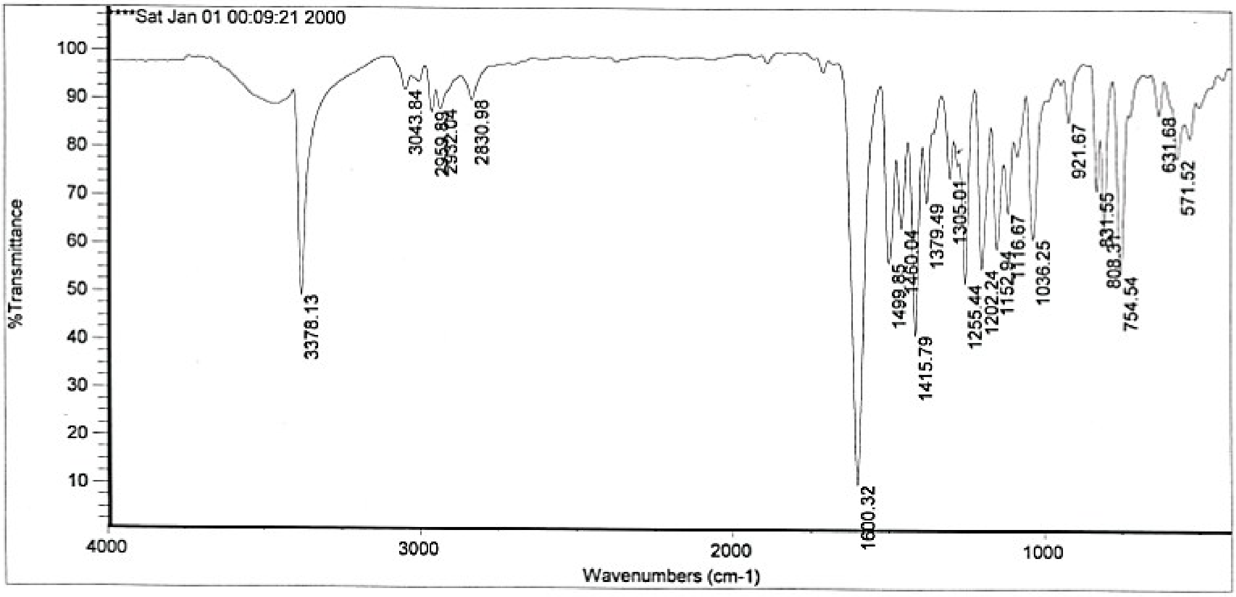

FT-IR spectrum of 2-(2, 4-dimethoxyphenyl)-2, 3-dihydro-1*H*perimidine

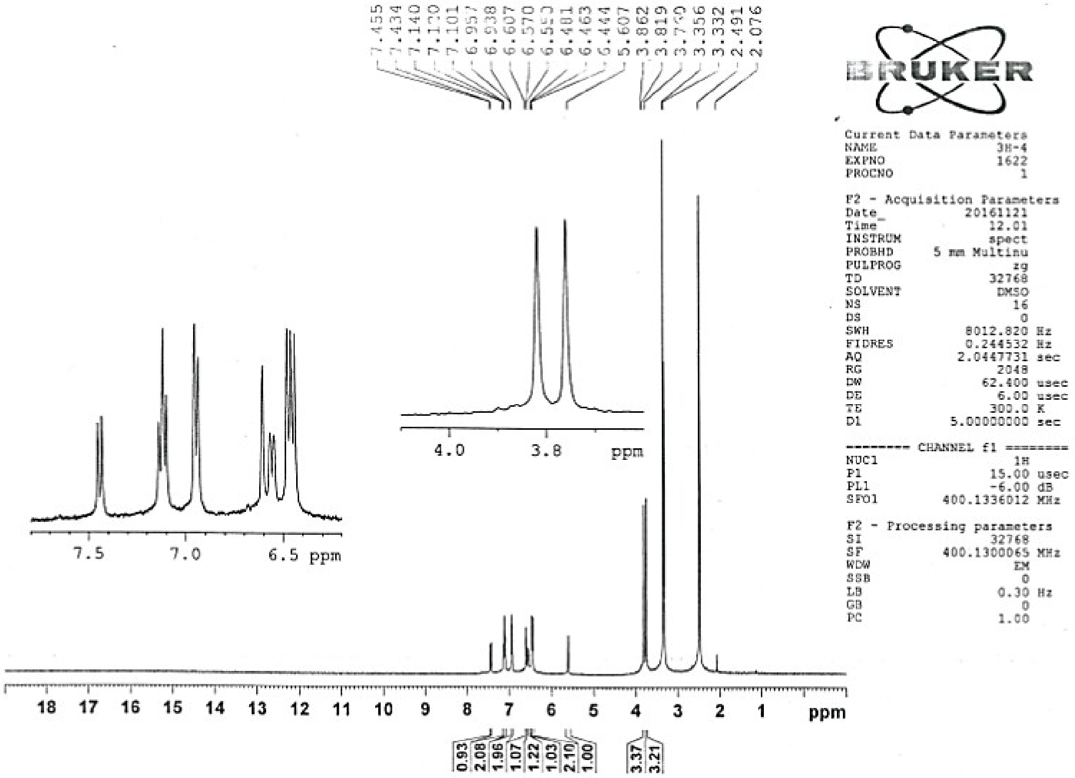

^{1}H NMR spectrum of 2-(2, 4-dimethoxyphenyl)-2, 3-dihydro-1*H*perimidine

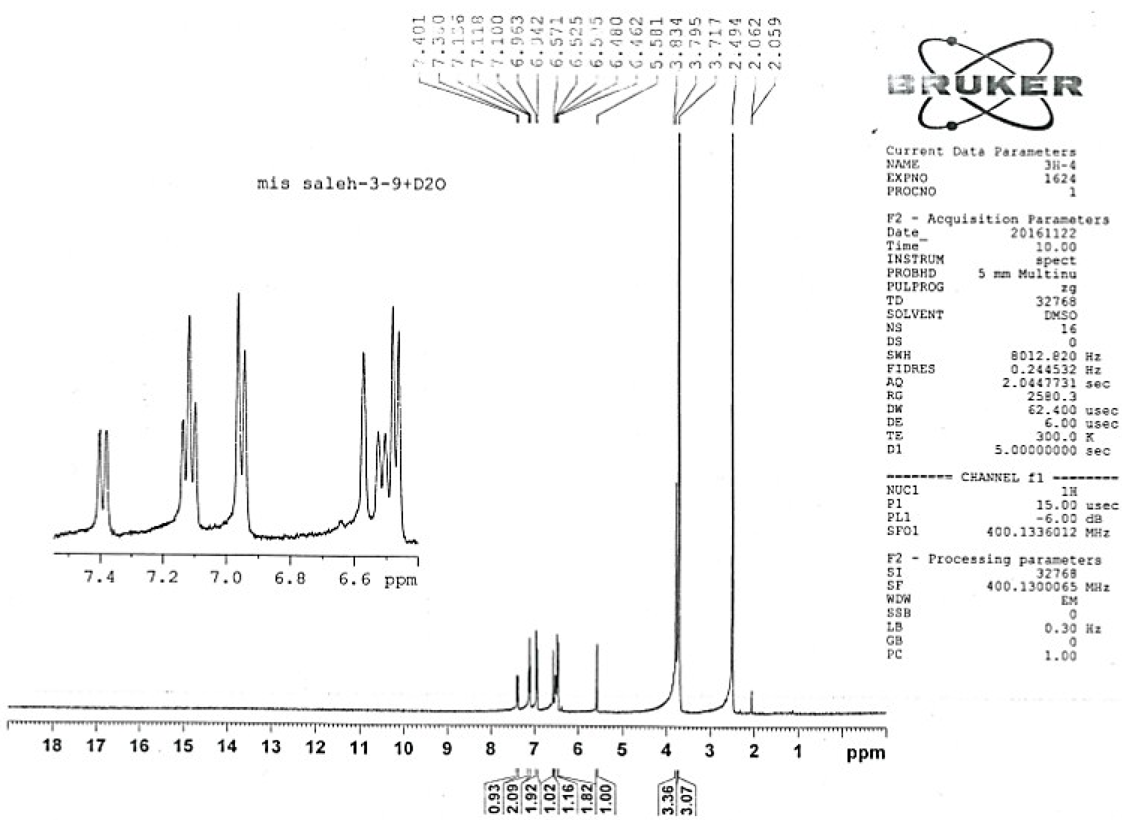

^{1}H NMR spectrum of 2-(2, 4-dimethoxyphenyl)-2, 3-dihydro-1*H*perimidine in the presence of D_2O

2-(2, 3-Dichlorophenyl)-2, 3-dihydro-1*H*perimidine (Table 2, 3i). IR (KBr): 3347, 3234, 3042, 2816, 1598, 1493, 1404 cm^{-1}.^{1}HNMR (DMSO, 400MHz): 3.35 (s, 1H, NH, exchange with D_2O), 5.77 (s, 1H, CH), 6.5 (d, 2H, *J*=6.8 Hz), 6.83 (s, 1H, NH, exchange with D_2O), 7.04 (d, 2H, *J*=8 Hz), 7.17 (m, 2H, *J*=7.2 Hz), 7.43 (m, 1H, *J*=7.6 Hz), 7.67 (d, 2H, *J*=6.8 Hz). ^{13}C NMR (DMSO):?

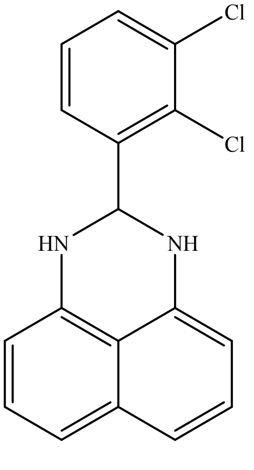

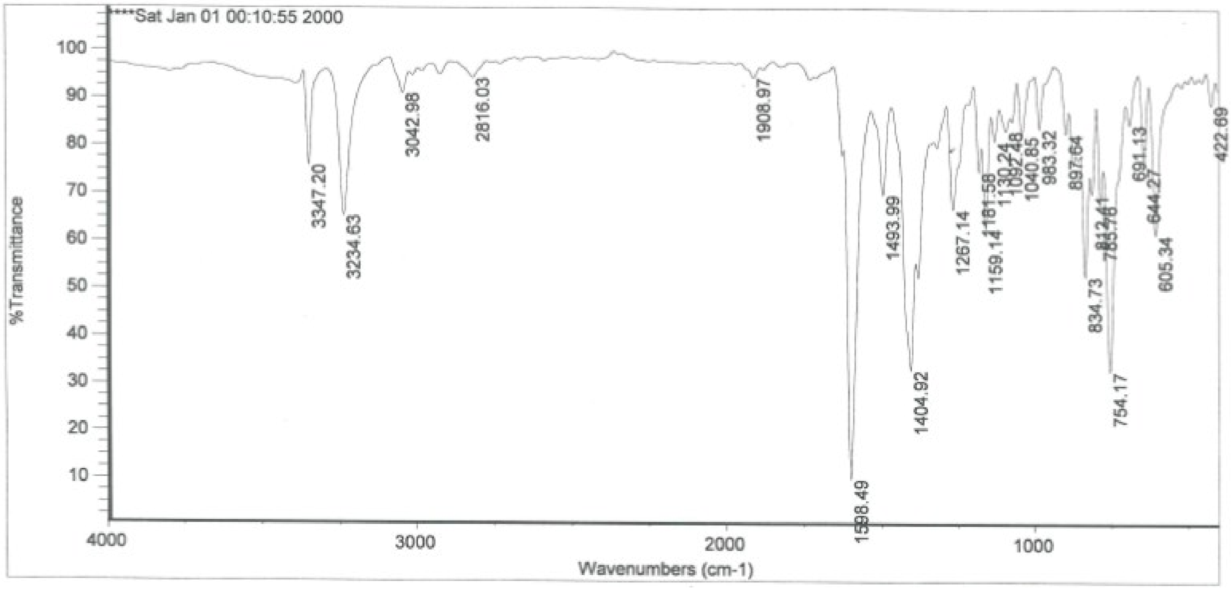

FT-IR spectrum of 2-(2, 3-Dichlorophenyl)-2, 3-dihydro-1*H*perimidine

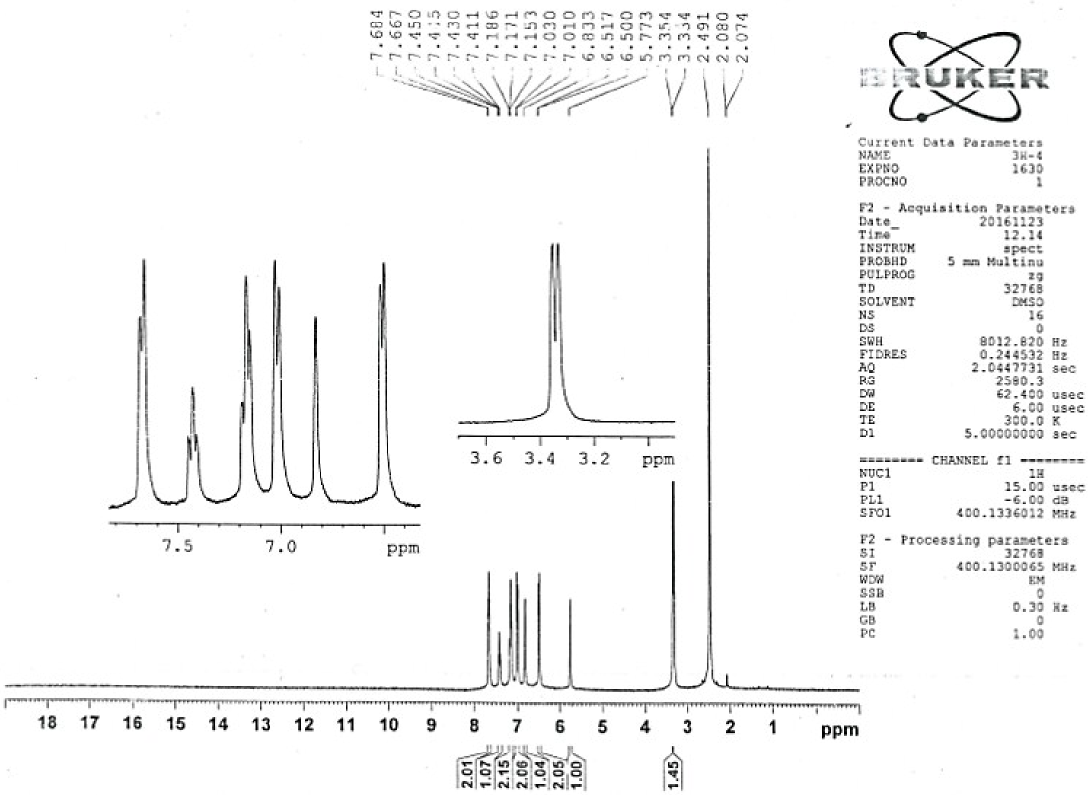

^{1}H NMR spectrum of 2-(2, 3-Dichlorophenyl)-2, 3-dihydro-1*H*perimidine

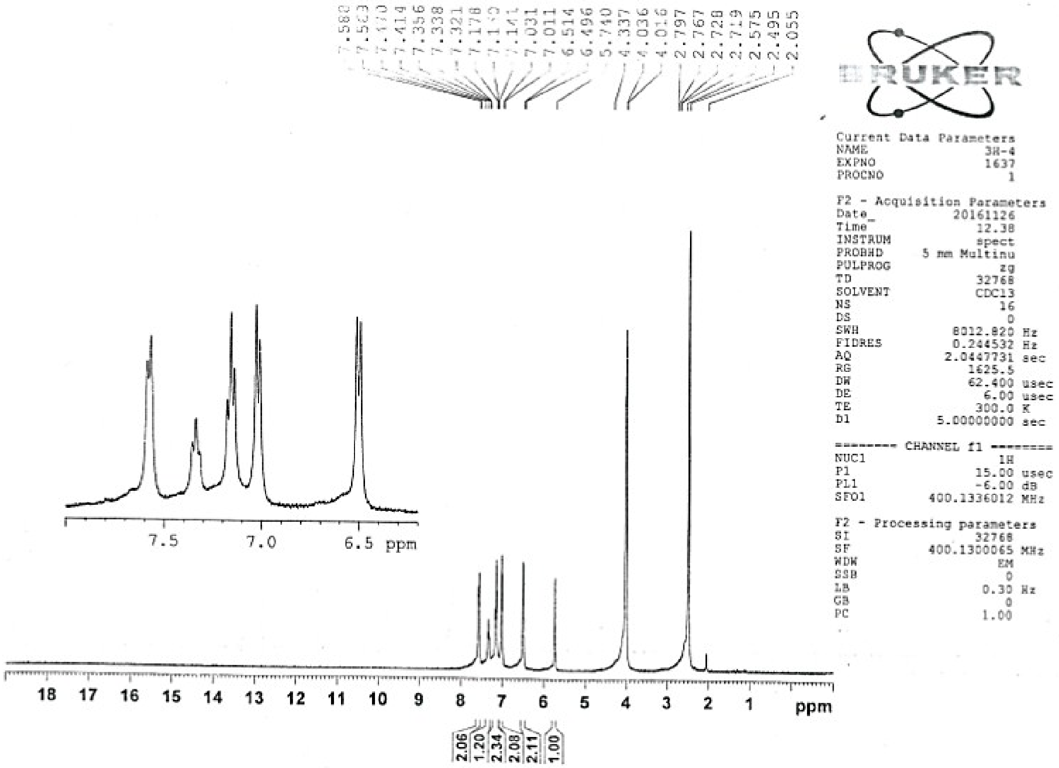

^{1}H NMR spectrum of 2-(2, 3-Dichlorophenyl)-2, 3-dihydro-1*H*perimidine in the presence of D_2O

2-(2, 3-Dimethoxyphenyl)-2, 3-dihydro-1*H*perimidine (Table 2, 3j). IR (KBr): 3360, 3045, 2932, 1595, 1480, 1422 cm^{-1}.^{1}HNMR (DMSO, 400MHz): 3,79 (s, 3H, CH_3), 3.82 (s, 3H, CH_3), 5.65 (s, 1H, CH), 6.48 (d, 2H, *J*=7.2 Hz), 6.55 (s, 2H, NH, exchange with D_2O), 6.97 (d, 3H, *J*=7.2 Hz), 7.05 (d, 2H, *J*=10 Hz), 7.12 (d, 2H, *J*=8Hz). ^{13}C NMR (DMSO):?

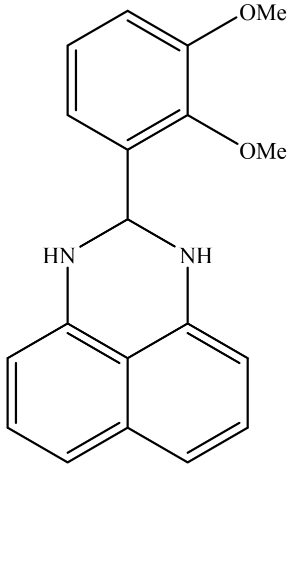

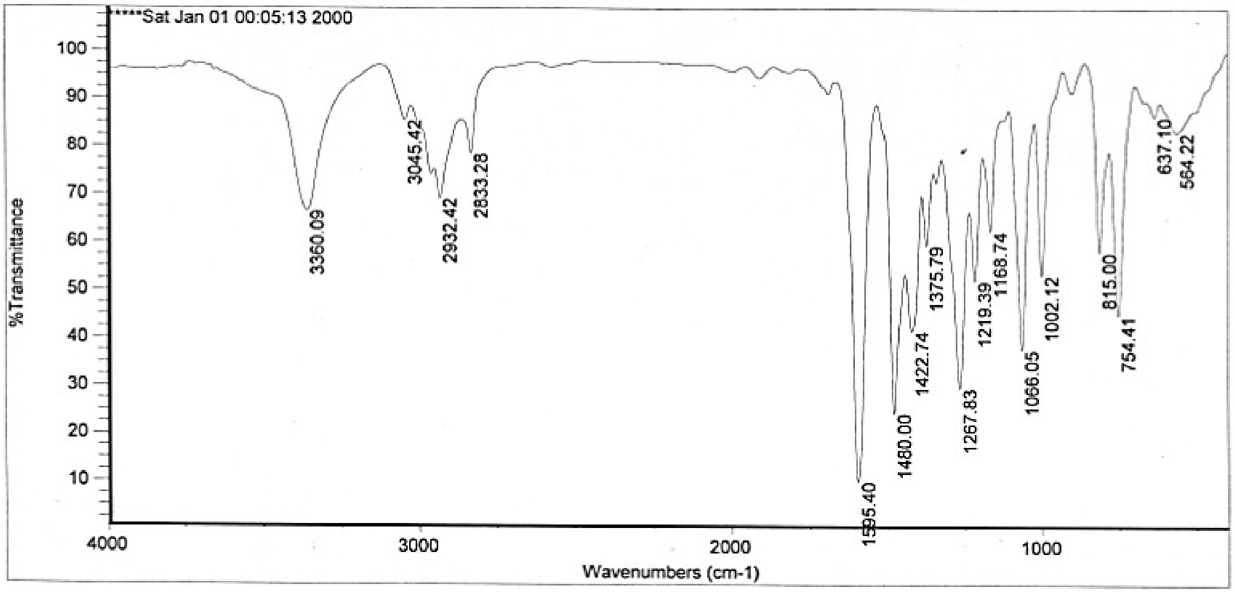

FT-IR spectrum of 2-(2, 3-dimethoxyphenyl)-2, 3-dihydro-1*H*perimidine

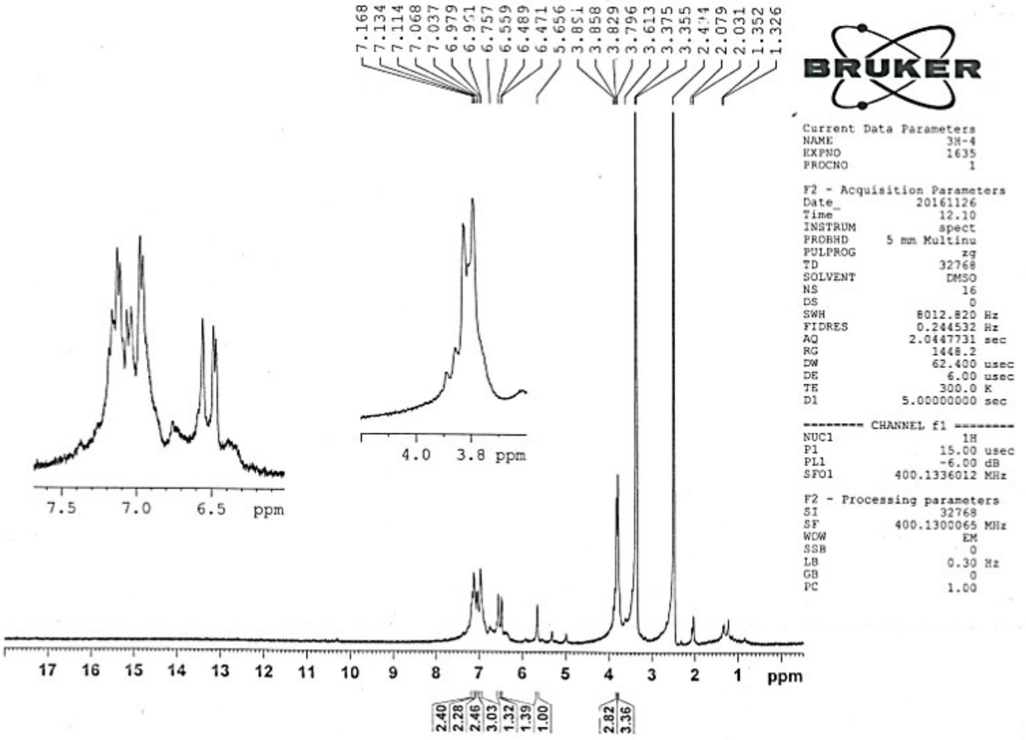

^{1}H NMR spectrum of 2-(2, 3-dimethoxyphenyl)-2, 3-dihydro-1*H*perimidine

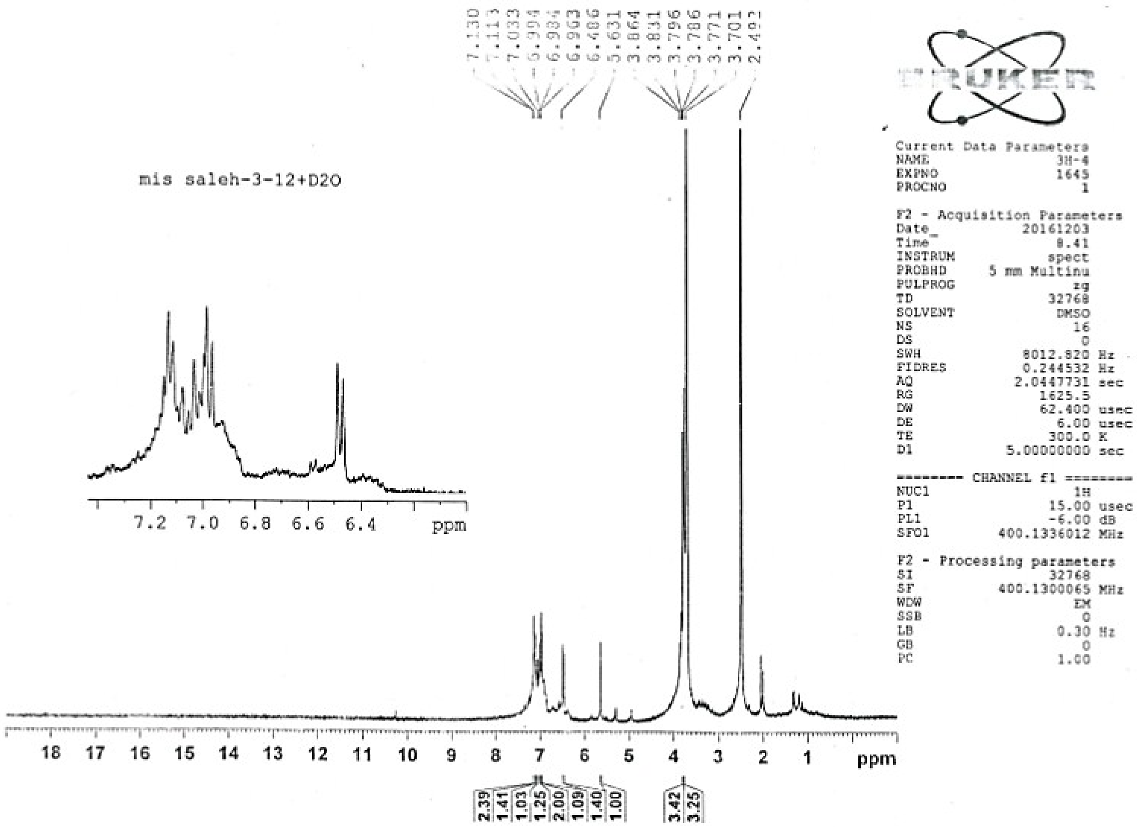

H NMR spectrum of 2-(2, 3-dimethoxyphenyl)-2, 3-dihydro-1*H*perimidine in the presence of D_2O

2-(3, 4-Dimethoxyphenyl)-2, 3-dihydro-1*H*perimidine (Table 2, 3k). IR (KBr): 3350, 2998, 2931, 1599, 1513, 1460, 1415 cm^{-1}. ^{1}HNMR (DMSO, 400MHz): 3,75 (s, 3H, CH_3), 3.76 (s, 3H, CH_3), 5.27 (s, 1H, CH), 6.47 (d, 2H, *J*=7.2 Hz), 6.66 (s, 1H, NH, exchange with D_2O), 6.97 (dd, 3H, *J*=7.2 Hz), 7.12 (dd, 4H, *J*=8 Hz), 7.2 (s, 1H, NH, exchange with D_2O) ^{13}C NMR (DMSO) [23] : 55.9, 56.5, 68.8, 104.8, 111.7, 113.0, 115.8, 120.7, 127.3, 134.5, 134.9, 143.8, 149.2, 149.59

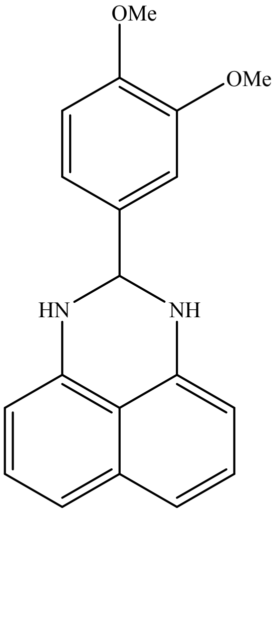

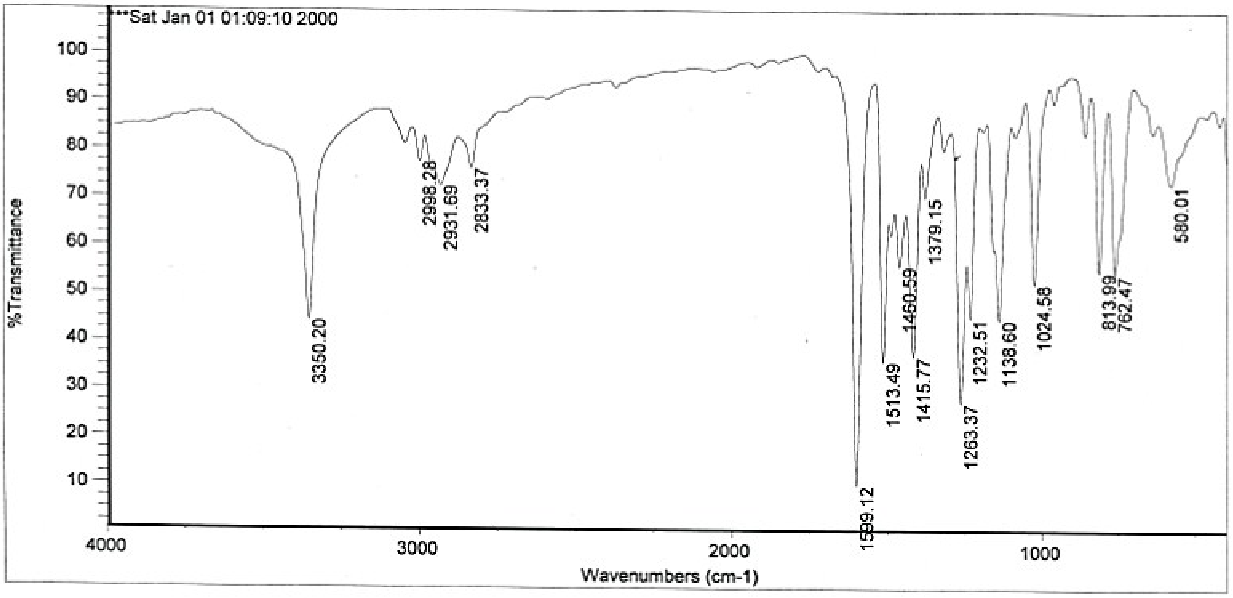

FT-IR spectrum of 2-(3, 4-dimethoxyphenyl)-2, 3-dihydro-1*H*perimidine

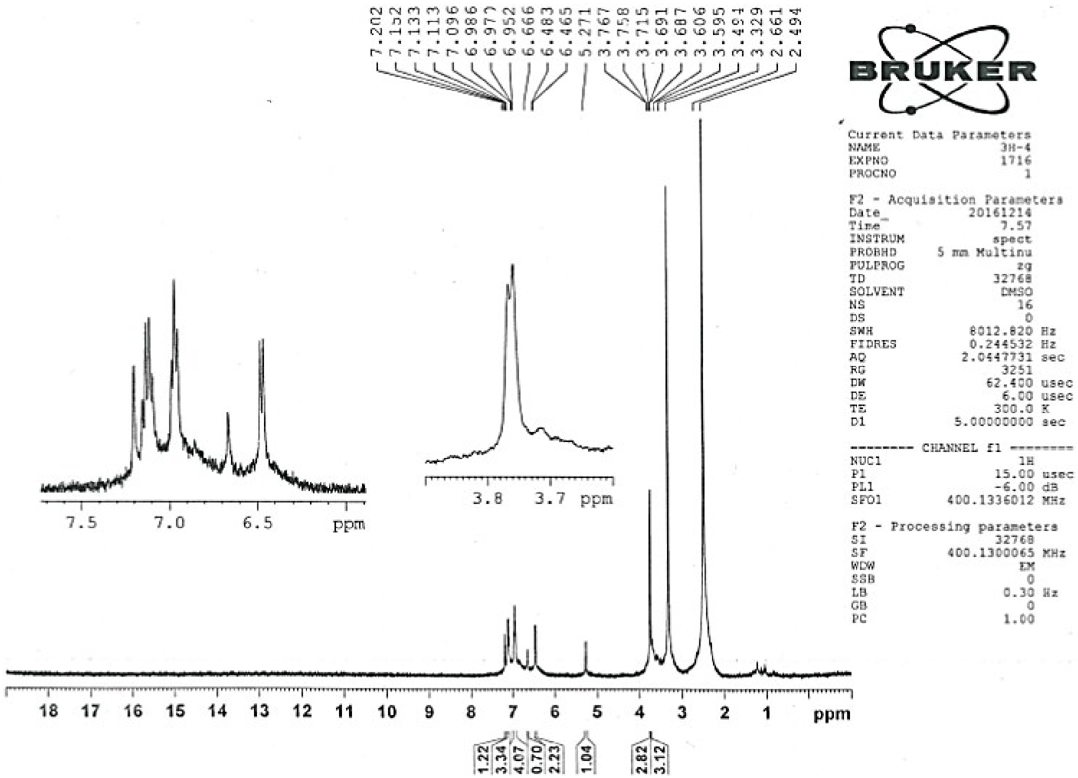

^{1}H NMR spectrum of 2-(3, 4-dimethoxyphenyl)-2, 3-dihydro-1*H*perimidine

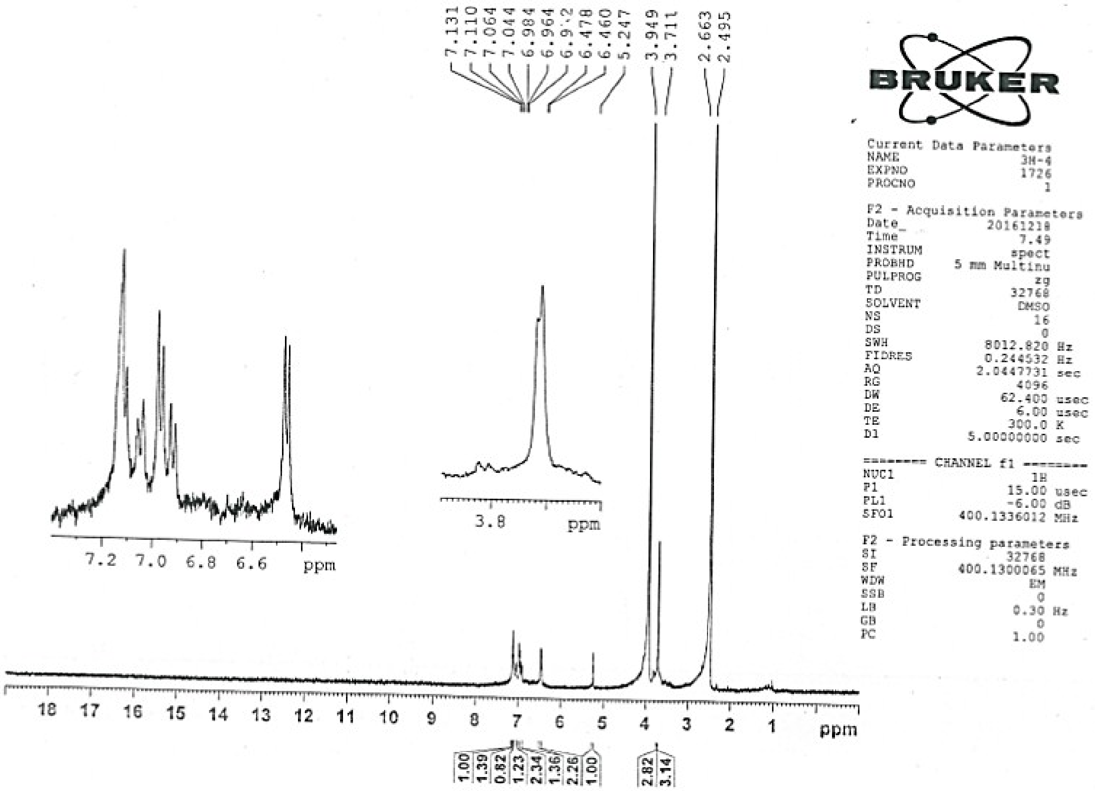

^{1}H NMR spectrum of 2-(3, 4-dimethoxyphenyl)-2, 3-dihydro-1*H*perimidine

in the presence of D_2O
